# PolyT-GNN: A Graph Neural Network Framework for Data-Driven Discovery of High-Temperature Two-Way Shape Memory Polymers

**DOI:** 10.1021/acsami.6c02689

**Published:** 2026-05-21

**Authors:** Amir Teimouri, Xiaowei Mu, Guoqiang Li

**Affiliations:** Department of Mechanical & Industrial Engineering, 5779 Louisiana State University, Baton Rouge, Louisiana 70803, United States

**Keywords:** graph neural network, graph representation learning, two-way shape memory polymer, transfer learning, machine learning

## Abstract

Two-way shape memory polymers (2W-SMPs) represent a promising class of smart materials capable of reversible actuation under cyclic thermal stimuli. Despite their potential for high-performance applications in aerospace, geothermal, self-healing, biomedical, and soft robotics, the discovery of new 2W-SMPs remains limited by the lack of large, systematic data sets and predictive design strategies, especially for high-temperature systems. Here, PolyT-GNN is introduced as a graph neural network framework for data-driven discovery of high-temperature 2W-SMPs. A curated data set of 170 experimentally validated 2W-SMPs is compiled from the literature. PolyT-GNN integrates atomic, bond, and molecular descriptors with explicit monomer weight ratios to accurately predict polymer transition temperatures, achieving a test *R* ^2^ of 0.84 even with limited data. Pretraining and fine-tuning together enhance prediction accuracy by about 38% compared to training from scratch, demonstrating strong cross-domain transferability. The framework further generates and screens over 80693 new 2W-SMP formulations, from which a polyethylene–dicumyl-peroxide system is synthesized and experimentally validated, showing a melting transition near 130 °C in close agreement with the predicted 113.25 °C. PolyT-GNN serves as a robust framework linking molecular structure, composition, and actuation behavior, enabling rational design of high-temperature two-way shape memory polymers for next-generation smart materials.

## Graphical abstract

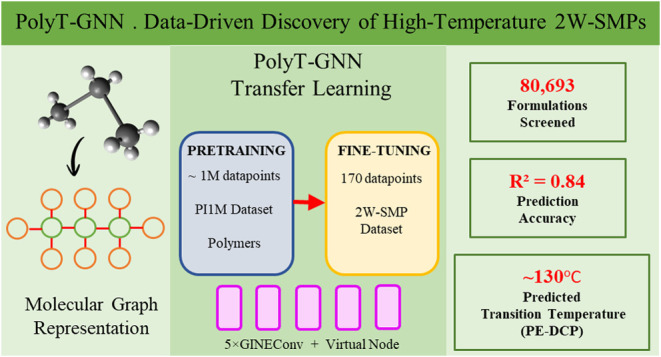

## 1 Introduction

Shape memory polymers (SMPs) are a class of stimuli-responsive materials capable of fixing a temporary shape and recovering their original configuration when exposed to external triggers such as heat, light, electricity, magnetic field, pH, and moisture.^1^ Their lightweight nature, tunable transition temperatures, and relatively low processing cost have made them attractive in diverse fields including geothermal energy harvesting,^2^ aerospace structures,^3^ soft robotics,^4^ self-healing,^5^ biomedical,^6^ and many other areas.^7^ Traditionally, most SMPs have been studied in the context of one-way actuation, where the material deforms into a temporary shape and recovers its permanent shape only once upon stimulation.^8,9^

A more advanced category, known as two-way shape memory polymers (2W-SMPs), extends this functionality by enabling reversible shape changes under cyclic stimuli. Unlike one-way SMPs that provide a single recovery event, 2W-SMPs can switch repeatedly between two shapes, offering continuous actuation without external reprogramming.^10^ This unique capability opens opportunities for adaptive systems,^11^ artificial muscles,^12^ deployable aerospace components,^13^ and self-regulating actuators.^14^ Nevertheless, the rational design of 2W-SMPs remains constrained by the inherently slow and complex experimental discovery process, the absence of systematic predictive models, and unresolved issues related to maintaining structural integrity and actuation stability under high-temperature conditions.

The reversible actuation behavior of 2W-SMPs depends on finely balanced molecular architectures—such as semicrystalline switching segments or liquid crystalline domains—that can undergo repeatable phase transitions.^15^ However, these phase transitions alone are not sufficient; the formation of a stable polymer network is essential to enable the storage of internal stress and preservation of chain orientation during cyclic actuation.^16^ In addition, the mechanical properties of the material—particularly modulus, elasticity, and deformability—play a critical role in governing the magnitude, efficiency, and durability of the two-way effect.^17^ Achieving such balance becomes even more complex at high temperatures, where stability and mechanical integrity are more difficult to maintain. One particularly demanding application that highlights these challenges is geothermal energy harvesting, where materials are exposed to superhot water environments exceeding 200 °C and must operate reliably under hydraulic pressure.^18–24^ In this context, 2W-SMPs can function as thermal switches or reversible proppants, expanding upon cooling to keep fractures open during cold water injection and contracting upon heating to regulate fluid flow once sufficient heat is absorbed.^2^ Such behavior directly enhances heat extraction efficiency and ensures sustainable operation of enhanced geothermal systems (EGS). Despite their promise, most reported 2W-SMPs lack the thermal stability and durability required for this extreme environment, highlighting a fundamental gap between existing material capabilities and the demands of high-temperature applications.

Although the design of two-way shape memory polymers remains challenging due to the complex coupling between molecular architecture, phase transitions, and internal stress evolution, several studies have provided important insights into the underlying mechanisms governing reversible actuation.^25–27^ Early work by Behl et al.^28^ demonstrated that fully reversible, stress-free two-way actuation can be achieved in multiphase polymer networks with two distinct crystallizable domains, where one defines the network geometry and the other acts as the actuator through reversible crystallization and melting. More broadly, two-way behavior has been shown to rely on (i) the presence of reversible phase transitions,^29^ (ii) the storage of internal stress through programmed chain orientation,^30^ and (iii) a stable network architecture capable of preserving this orientation during cyclic thermal loading.^31^ The resulting cyclic deformation arises from thermally driven phase transitions of oriented chain segments, governed by the interplay between entropy elasticity and crystallization-induced structural changes. Subsequent studies on liquid crystalline elastomers, such as Liquid crystalline elastomers as artificial muscles and flexible actuators, highlighted the role of mesogen alignment and nematic–isotropic phase transitions in enabling repeatable actuation.^32,33^ In addition, hybrid systems combining multiple switching domains have been explored to enhance actuation stability and reversibility, as reported in Stress-free two-way shape memory polymer networks based on dual crystalline domains.^34^ While these studies establish fundamental design concepts, they are typically limited to specific material systems and do not provide generalized, composition-aware design rules applicable across diverse polymer chemistries.

This limitation is further compounded by the scarcity of large, systematic data sets and predictive modeling frameworks for 2W-SMPs. In contrast, one-way SMPs have been extensively studied, with comparatively larger data sets and well-established correlations between molecular structure, transition temperature, and shape recovery performance.^35–38^ As a case in point, Yan et al.^39^ employed convolutional neural networks to identify 14 novel thermoset one-way shape memory polymers (1W-SMPs), achieving enhanced recovery stress compared to conventional approaches. Moreover, Ibarra et al.^40^ developed deep neural network for modeling shape memory polymers using time, temperature, and stress inputs. These studies demonstrate how data-driven methods can accelerate the design of 1W-SMPs by uncovering hidden structure–property relationships. However, comparable advances have not yet been realized for 2W-SMPs. Current research on 2W-SMPs remains constrained by small and fragmented data sets and the lack of comprehensive machine-learning frameworks, which underscores the need for advanced data-efficient learning strategies to enable predictive modeling and rational design.

In recent years, deep learning and graph-based frameworks have further advanced polymer property prediction by eliminating the need for manual feature engineering. Models such as graph convolutional networks (GCNs),^41–43^ message passing neural networks (MPNNs),^44,45^ and multitask graph neural networks (GNNs)^46–48^ have demonstrated remarkable success in predicting polymer properties. However, despite their superior representational capacity, most of these deep learning models require large, well-labeled data sets to achieve high accuracy and generalizability.^49,50^ In polymer science—particularly for emerging systems such as 2W-SMPs—experimentally validated data remain scarce and highly fragmented, making it challenging to train conventional deep learning models effectively.

To address the challenges posed by limited data availability, recent studies have explored data-efficient representation learning strategies such as representation learning, transfer learning, and self-supervised graph neural networks.^50–52^ Transfer learning and self-supervised GNN frameworks have demonstrated strong potential in polymer informatics, where pretraining tasks such as node masking, edge prediction, or graph contrastive learning allow models to extract chemically meaningful features directly from molecular structure.^53^ Recently, Jiang et al.^54^ developed a property-guided variational autoencoder framework capable of generating polymers with complex topologies and desired structural properties, demonstrating the effectiveness of unsupervised graph-based models in capturing polymer connectivity and topology–property relationships. Similarly, the PolyCL framework^55^ employed explicit and implicit contrastive learning augmentations to enhance polymer graph representations, further illustrating the promise of self-supervised approaches in achieving robust feature learning from scarce data. These approaches collectively enable the development of predictive models that remain robust and accurate even when the number of experimentally measured data points—such as transition temperatures of 2W-SMPs—is limited.

To overcome the limitations of current experimental trial-and-error approaches, a predictive framework leveraging GNNs was developed to discover 2W-SMPs capable of operating at elevated temperatures. The model was first pretrained on the large-scale PI1M polymer data set, enabling it to learn general chemical and topological representations from about one million polymer graphs. It was then finetuned on a curated, high-quality data set of experimentally validated 2W-SMPs, allowing domain adaptation to the specific chemistry and actuation behavior of 2W-SMP systems. By representing each polymer as a molecular graph enriched with atomic, bond-level, and mixture-level features, including the explicit incorporation of monomer weight ratios, the proposed PolyT-GNN captures both local connectivity and global compositional effects. Beyond achieving accurate prediction of transition temperatures, PolyT-GNN was further employed to generate and screen previously unseen 2W-SMP formulations that were not included in the training data set, from which a promising candidate was selected for synthesis and experimental validation. To the best of our knowledge, this study presents the first graph neural network approach tailored for 2W-SMPs that explicitly incorporates monomer weight ratios, thereby enabling both predictive modeling and generative discovery of high-temperature formulations.

## 2 Methods

### 2.1 2W-SMP Data Set: Curation and Quality Control

To enable effective model pretraining and mitigate the limitations of the small 2W-SMP data set, the PI1M polymer data set was utilized—a large-scale collection comprising approximately one million unique polymer repeat units. This data set was developed by Ma et al.^56^ through the training of a generative model using polymer data sourced from the PolyInfo database.^57^ The data set covers a broad chemical space that includes a diverse range of homopolymers, copolymers, and cross-linked architectures, providing a rich source of unlabeled structural information for unsupervised or self-supervised pretraining.

To establish a framework for discovering 2W-SMPs capable of actuating and maintaining stability at elevated temperatures, a data set was curated from values reported in the literature. Table S1 indicated the specific checks criteria evaluated in publications prior to extracting the transition temperature of the 2W-SMPs. Since the actuation mechanism of 2W-SMPs depends strongly on the type of switching domain, the relevant transition temperature was selected accordingly.

For semicrystalline 2W-SMPs, reversible actuation originates from crystallization and melting, where elongation occurs upon crystallization during cooling and contraction takes place upon melting during heating.^58^ When the temperature is above melting temperature (*T*_m_), some cross-linked semicrystalline 2W-SMPs can further demonstrate reversible actuation, due to the rubbery elasticity.^58^ Between these two transitions (*T*_m_ and *T*_c_ (crystallization temperature)), *T*_m_ is the more critical parameter, as it defines the upper operational limit of true reversible actuation, i.e., reversible actuation without an external tensile force,^58^ and determines whether the material can survive and function under high-temperature conditions. Although the *T*_c_ also plays a role in governing cyclic behavior, it is highly sensitive to processing conditions and is less consistently reported in the literature. Therefore, in constructing the data set, Tm values were collected as the primary transition temperature for semicrystalline systems, ensuring a consistent and application-relevant property for model development.

For liquid crystalline 2W-SMPs, the key transition is the nematic–isotropic transition temperature (*T*_ni_). In the nematic state, liquid crystalline domains exhibit orientational order, and actuation arises from the collective alignment or misalignment of these mesogens under thermal cycling.^59^ When the material is heated above *T*_ni_, this order is lost and the polymer transitions into an isotropic state, typically resulting in contraction; upon cooling below *T*_ni_, nematic ordering is restored, inducing expansion. Accordingly, *T*_ni_ was selected as the defining transition temperature for liquid crystalline 2W-SMPs, since it directly governs the reversible ordering and actuation behavior of these systems.

The data was compiled into a data set utilized for training our machine learning model. 170 data points have been added to the data set. The complete data set in Excel format, including the monomer/cross-linker names, Simplified Molecular Input Line Entry System (SMILES) strings, weight ratios, and corresponding transition temperatures, is publicly available at https://github.com/faria-tm/PolyT-GNN. The data set encompasses diverse classes of 2W-SMPs—including semicrystalline, amorphous, and liquid crystalline systems—covering a broad spectrum of chemical structures and switching mechanisms. The distribution of transition temperatures across the data set is shown in Figure S1. From Figure S1, it is seen that the majority of the 2W-SMPs have transition temperature in the range of 40 °C–60 °C, only a few of them have transition temperatures higher than 100 °C.

### 2.2 Graph-Based Encoding of Molecular Structures

SMILES is a widely used notation in cheminformatics that encodes chemical structures as linear text strings.^60^ This representation translates the connectivity of atoms, bond types, ring closures, and branching into a standardized and machine-readable format, enabling efficient storage, exchange, and computational processing of molecular information. However, while SMILES strings are convenient for storage and parsing, they do not directly expose the relational structure of molecules required for graph-based learning. To bridge this gap, SMILES representations are converted into molecular graphs *G* = (*V*, *E*), where nodes *v*_*i*_ ∈ *V* correspond to atoms and edges *e*_*ij*_ ∈ *E* represent chemical bonds. Each edge *e*_*ij*_ connects a pair of nodes *v*_*i*_ and *v*_*j*_, thereby preserving the atomic connectivity within the molecular structure. Each node and edge can then be enriched with physicochemical attributes, providing a structure-aware input representation that is particularly well suited for graph neural networks.

Atomic-level features provide a detailed representation of the local chemical environment, allowing the model to capture variations in bonding, connectivity, and functional group chemistry. These features were extracted from each monomer using RDKit. All atomic-level features and their descriptions are summarized in Table S2. Fourteen atomic features were extracted, and redundant features were identified and removed based on Pearson correlation analysis. The Pearson correlation coefficient between two features *x* and *y* is defined as

1 $$r_{\mathrm{xy}}=\frac{\sum_{i=1}^{n}\left(x_{i}-\mathrm{x̅}\right)\left(y_{i}-\mathrm{y̅}\right)}{\sqrt{\sum_{i=1}^{n}{\left(x_{i}-\mathrm{x̅}\right)}^{2}}\sqrt{\sum_{i=1}^{n}{\left(y_{i}-\mathrm{y̅}\right)}^{2}}}$$

where *x̅* and *y̅* denote the mean values of the features. Low-correlation features (|*r*_*xy*_| > τ, with threshold τ = 0.01) were excluded to reduce redundancy and avoid multicollinearity in the model inputs. Four features with the highest correlation—aromaticity (IsAromatic), ring membership (IsInRing), partial atomic charge (PartialCharge), and number of implicit hydrogens (NoImplicitHs)—were selected for the downstream task. Figure S2 indicated the Pearson correlation of the 14 atomic features.

The selection of these four features is chemically meaningful in the context of transition temperature prediction. Aromaticity is closely linked to molecular rigidity and π–π stacking interactions, both of which strongly influence thermal stability and the glass/melting transitions of polymers.^61^ Ring membership similarly affects conformational flexibility; cyclic structures often impose steric restrictions that raise transition temperatures compared to their acyclic counterparts.^62^ Partial atomic charge reflects the electronic distribution within the molecule and governs intermolecular interactions such as hydrogen bonding and dipole–dipole forces, which are critical in determining phase-transition behavior.^63^ Finally, the number of implicit hydrogens provides information about the degree of substitution on atoms, influencing both steric environment and local electronic effects. Together, these features capture structural rigidity, electronic distribution, and intermolecular interaction potential, offering a chemically consistent rationale for their strong correlation with polymer transition temperatures.

Molecular-level descriptors provide a global representation of structural and physicochemical properties, complementing the local atomic features by capturing characteristics such as size, polarity, and topology. At this level, a set of valid physicochemical descriptors was identified from the RDKit library, with only those that consistently returned valid numerical values across all monomers retained to ensure data set integrity. All molecular-level features and their descriptions are summarized in Table S3. Initially, 160 molecular features were extracted using RDKit, and Pearson correlation analysis was employed to identify 11 features with strong correlation to the transition temperature. Figure S3 illustrates the Pearson correlation matrix among the selected molecular descriptors (threshold τ = 0.24) and their relationship with the transition temperature.

Bond information was encoded through bidirectional edges, where each covalent bond between atoms was represented by two directed edges (*v*_*i*_ → *v*_*j*_ and *v*_*j*_ → *v*_*i*_). This design ensured that message passing within the graph neural network was symmetric, allowing information to flow consistently in both directions along a bond. In addition to bidirectionality, each edge was further enriched with bond-specific features, including one-hot encoding of bond type (single, double, triple, aromatic), aromaticity, conjugation state, and the electronegativity difference between bonded atoms. These descriptors capture critical aspects of chemical bonding that strongly influence polymer transition temperatures. Bond order and aromaticity affect chain rigidity and thermal stability,^64,65^ while conjugation contributes to electron delocalization, impacting both intermolecular interactions and energy dissipation during phase transitions.^66^ The electronegativity difference provides a measure of bond polarity, which governs dipolar interactions and hydrogen bonding tendencies, further shaping the thermal response of polymers.^67^ Electronegativity difference calculation based on Pauling values (Table S4) is described in Section S1.1.

For each 2W-SMP entry, a molecular graph was constructed from the SMILES strings of its constituent monomers and their corresponding weight ratios. An example of creating the graph representation of a 2W-SMP is illustrated in Figure 1. From each monomer, atomic features were extracted to serve as node attributes. When constructing graphs for 2W-SMPs made of two or more monomers, the atoms of different monomers often share similar chemical environments (for example, two monomers may both contain carbonyl groups, aromatic carbons, or hydroxyl oxygens). If only generic atomic features (atomic number, degree, aromaticity, etc.) are used, the model cannot easily distinguish to which monomer a given atom belongs. This creates ambiguity, especially since the monomer ratios are critical for determining the thermal behavior of 2W-SMPs. To resolve this issue, a monomer identifier is appended as an additional feature to every atom. In Figure 1, atomic features belonging to Monomer A are labeled with the identifier “1,” while those from Monomer B are labeled with “2”. This additional feature ensures that during message passing in the GNN, the model accounts not only for the local chemical environment of each atom but also for its monomeric origin. By explicitly distinguishing atoms from different monomers, the representation captures the compositional context of the monomer mixture, thereby enabling more accurate learning of structure–property relationships.

**Figure 1:**
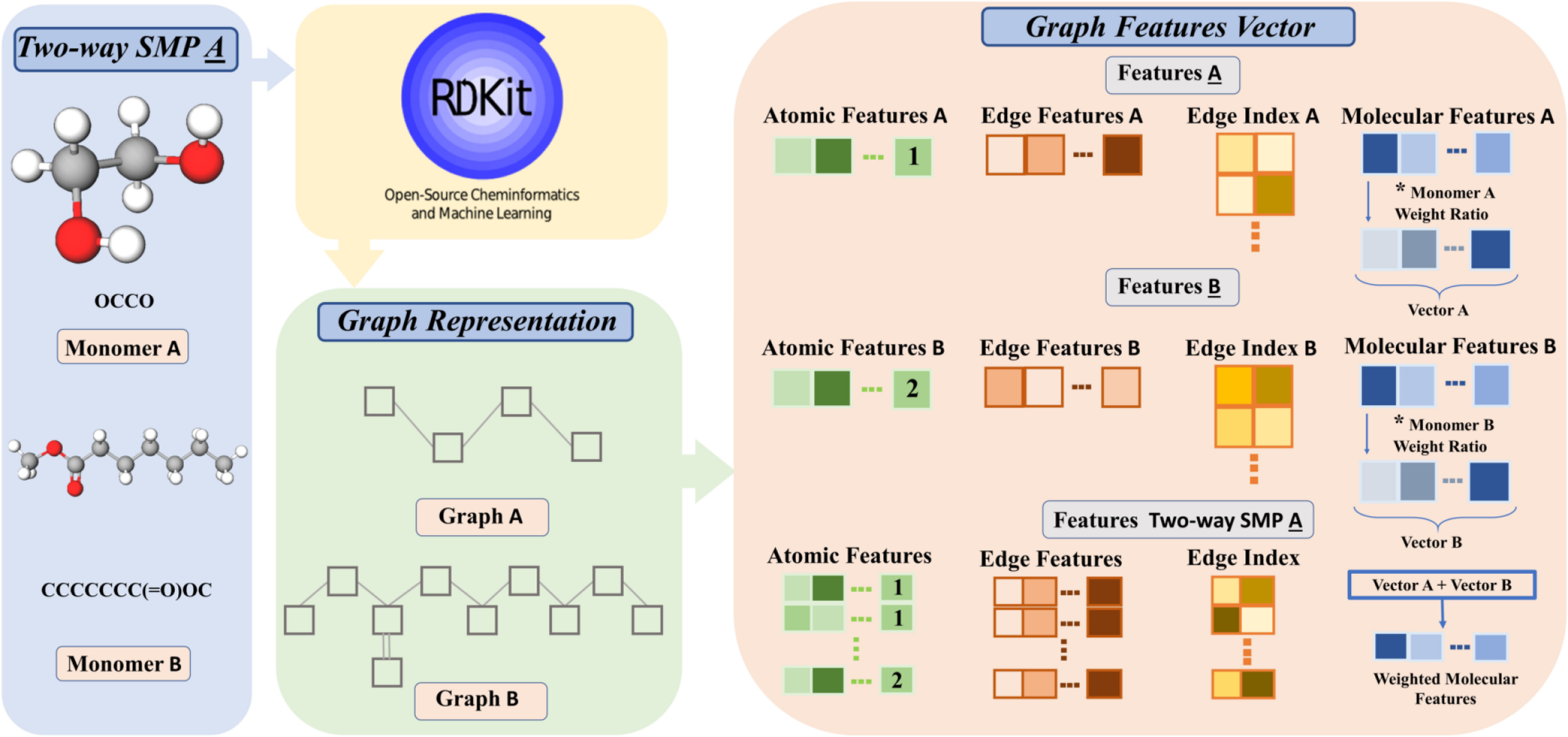
Schematic of the feature construction process for a 2W-SMPs (Two-way SMP A) composed of two monomers. Left panel: The chemical structures and SMILES strings of Monomer A (top) and Monomer B (bottom). Middle panel (Graph Representation): Graph A and Graph B illustrate the connectivity of Monomer A and Monomer B, respectively, with each square denoting an atom node and each line denoting a bond edge. Right panel (Graph Features Vector): Atomic features (green); Edge features (orange); Edge index (yellow); and Molecular descriptors (blue). Each molecular descriptor vector is weighted according to the reported monomer ratio, yielding Vector A and Vector B. These vectors are then combined to form the weighted molecular features of Two-way SMP A.

In addition, bond-level information was incorporated by defining an edge index (capturing connectivity between atoms) along with edge features. In parallel, molecular descriptors were computed for each monomer and aggregated according to the experimentally reported molar weight ratios, yielding a weighted feature vector that captured the global physicochemical characteristics of the polymer mixture. The resulting graph representation therefore contained four integrated components: (i) atom-level node features, (ii) bond-level edge features, (iii) bond-derived edge connectivity, and (iv) monomer mixture-level molecular descriptors. An illustrative example of the feature construction process for a representative monomer is provided in the Supporting Information (Figure S4). Finally, the transition temperature (*T*_m_ or *T*_ni_, depending on the system) was assigned as the target property for supervised training. This procedure ensured that both local chemical compositions and global environmental effects were systematically incorporated into the predictive framework.

Recent studies have introduced a range of encoding strategies for copolymer systems, including sequence-based, graph-based, and data-driven representation learning approaches that aim to capture polymer connectivity and architecture. For instance, the physics-guided framework^68^ represents polymers at the repeat-unit level while incorporating architectural information, whereas the multimodal representation learning approach^69^ exploits large-scale data sets to learn transferable polymer embeddings. In addition, the periodic polymer graph representation^70^ explicitly encodes polymer connectivity by enforcing periodic boundary conditions within graph structures. While these approaches have demonstrated strong predictive performance, they primarily focus on idealized repeat-unit representations or single-chain architectures and do not explicitly account for compositional effects in multicomponent systems.

In contrast, the proposed encoding scheme in this work is specifically designed for multicomponent 2W-SMP systems by (i) explicitly incorporating monomer composition and weight ratios, enabling direct modeling of mixture-dependent behavior, (ii) integrating atomic-, bond-, and molecular-level features within a unified graph-based framework to capture both local chemical environments and global physicochemical properties, and (iii) ensuring permutation invariance through the aggregation of monomer embeddings, which guarantees consistent predictions regardless of monomer ordering. These features allow the proposed approach to more effectively capture the complex structure–composition–property relationships governing two-way shape memory polymers.

### 2.3 Pretraining–Fine-Tuning GNN Pipeline

The discovery of 2W-SMPs capable of operating at elevated temperatures is hindered by the scarcity of high-quality, labeled experimental data. Traditional supervised learning models, such as support vector machines and gradient boosting regressors, depend on large, curated data sets to achieve reliable predictions; yet, fewer than two hundred 2W-SMP formulations have been reported in the literature, limiting their ability to generalize effectively. To overcome this constraint, a two-stage learning strategy was adopted, combining large-scale self-supervised pretraining on a broad polymer corpus with targeted fine-tuning on a curated 2W-SMP data set. This hierarchical approach enables the model to first capture universal polymer chemistry from a diverse unlabeled database and then specialize in the structural and functional characteristics that govern reversible actuation at high temperatures.

Recent advances in polymer representation learning have shown that GNNs can learn transferable embeddings that encode atomic connectivity, bonding environments, and global physicochemical features.^71,72^ Building on this foundation, the Graph Isomorphism Network with edge features (GINEConv) was employed as the backbone of the two-stage framework, hereafter referred to as PolyT-GNN, where “*T*” denotes the transition temperature, the key target property governing the thermally driven actuation behavior of two-way shape memory polymers. GINEConv extends the expressivity of standard message-passing networks by jointly leveraging atomic and bond attributes, allowing the encoder to distinguish subtle chemical variations that affect transition temperature.^73^ In the pretraining stage, the model learns chemically meaningful representations from a large unlabeled polymer database through multitask self-supervision; during fine-tuning, these representations are adapted to the smaller 2W-SMP data set to yield task-specific embeddings for monomers and cross-linkers that serve as transferable fingerprints for downstream property prediction.

Figure 2 illustrates the PolyT-GNN encoder architecture and message-passing workflow described below. The PolyT-GNN encoder is pretrained on a large unlabeled corpus (≈1 million polymer SMILES from PI1M) to learn chemistry-aware graph representations before any task-specific supervision. This follows the general motivation in polymer representation learning that large unlabeled data sets can endow downstream models with transferable structure–property priors. Each SMILES is converted to a molecular graph. The selected atomic, bond, and global molecular features defined in Section 2.2 are computed with RDKit and then scaled using min–max normalization, ensuring that all values were mapped to the range [0,1]. The transformation is defined as

2 $$X^{\prime }=\frac{X-X_{\min}}{X_{\max}-X_{\min}}$$

where *X* denotes any feature (node, edge, molecular descriptor, or target label), *X*_min_ and *X*_max_ are the per-feature minima/maxima computed on the training set, and *X*′ is the min–max–normalized value.

**Figure 2:**
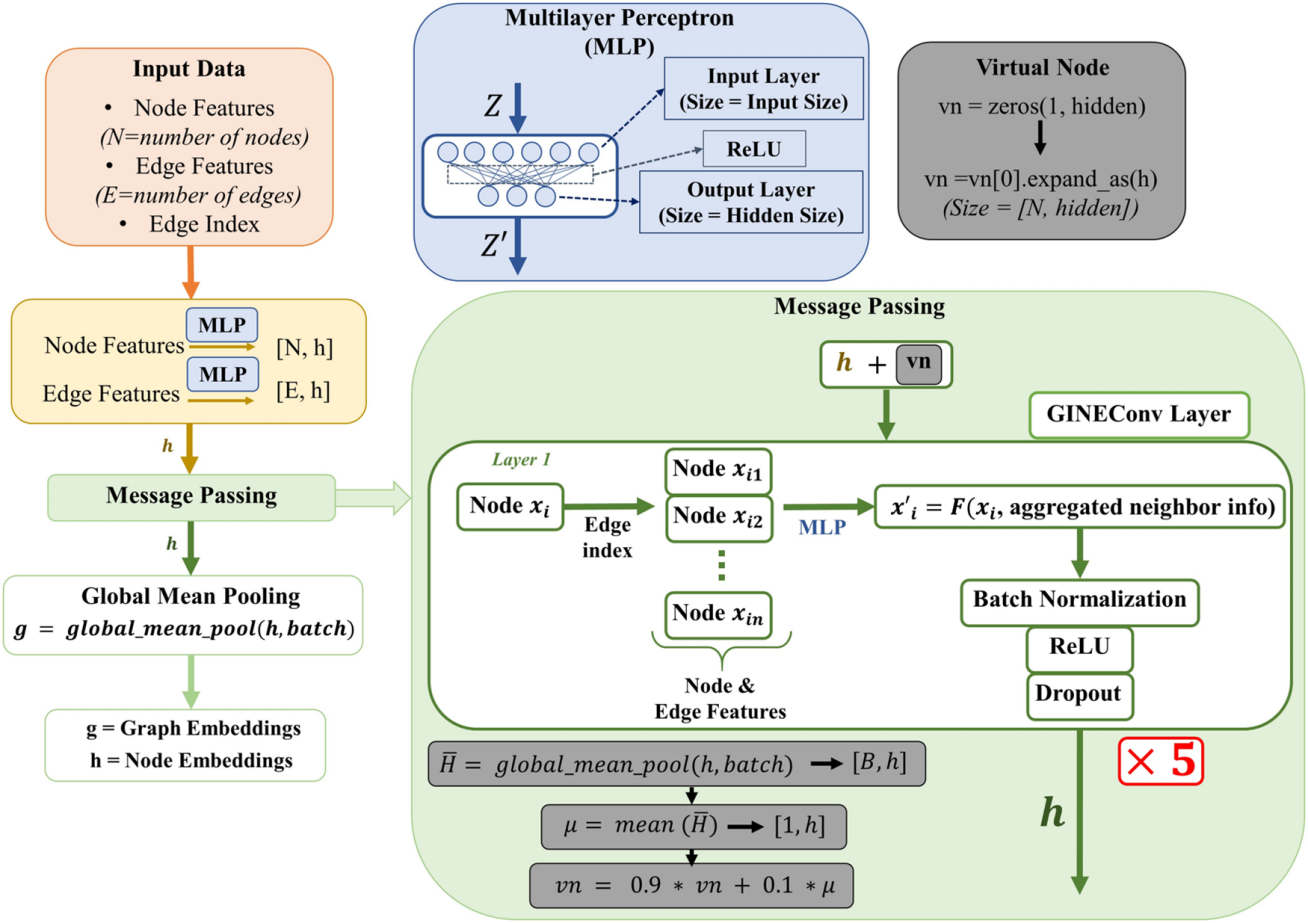
Schematic illustration of the PolyT-GNN encoder architecture based on the Graph Isomorphism Network with edge features (GINEConv). The encoder receives atomic-level (node) and bond-level (edge) features derived from each polymer SMILES, together with an edge index that defines the connectivity list of bonded atom pairs in the molecular graph. Node and edge features are first projected into latent vectors of dimension h through multilayer perceptrons (MLPs). Message passing is performed through five stacked GINEConv layers, where each node *x*_*i*_ updates its hidden state *x*_*i*_^′^ = *F*(*x*_*i*_, aggregated neighbor info) using both neighboring atom and bond information. A virtual node (vn)—a learnable vector appended to each graph—is included to capture global chemical context and facilitate long-range message propagation. The virtual node is updated after each layer via an exponential moving average of the pooled node embeddings *H̅*. Finally, global mean pooling, an averaging operation that combines all node embeddings within a molecule, produces a single graph-level representation *g* that encodes the overall polymer structure for downstream tasks.

The encoder comprises five GINEConv blocks (hidden width 64), each block is followed by batch normalization, ReLU activation, and dropout (0.3) regularization. Data were split into 85/15 train–test partitions, and mini-batches were formed with a size of 256 for pretraining. A virtual node exchanges global context with all atoms; its state is updated after every block using an exponential moving average of mean-pooled node features (0.9 old, 0.1 new). Node embeddings are aggregated by global mean pooling to yield a graph embedding *g* ∈ *R* ^64^; the width (64) was selected via a latent-size sweep—see Table S5.

Incorporating a virtual node significantly enhances the expressive power of the PolyT-GNN encoder (See Table S6). Traditional message-passing networks propagate information only through local neighborhoods, limiting the ability of each atom to access long-range chemical context—an important factor in polymers where distant substructures, cross-linking patterns, and mixture-level interactions collectively influence macroscopic properties. The virtual node introduces a global learnable state that communicates with every atom at each GINEConv layer, enabling efficient propagation of graph-level information and facilitating long-range dependency learning. Recent studies have demonstrated similar benefits of virtual-node–augmented GNNs in other scientific domains; for example, Okabe et al. and Qian et al. showed that virtual nodes substantially improve long-range information transfer and boost predictive accuracy across physics-driven graph learning task.^74,75^

PolyT-GNN comprises the GINE encoder followed by three lightweight heads that implement a multitask, self-supervised objective (Figure 3). Given a polymer graph, the encoder produces node embeddings *h* and a graph embedding *g*. These representations feed three parallel heads:

1. Descriptor head (graph-level). An MLP maps *g* to a vector of RDKit molecular descriptors, yielding *d̂*. The loss is mean-squared error (eq 3) against the normalized descriptor targets *d*.
3 $$L_{\mathrm{desc}}=-\frac{1}{B}\sum_{b\in B}\frac{1}{R}{a∥{\mathrm{d̂}}^{\left(b\right)}-d^{\left(b\right)}a∥}_{2}^{2}$$
  Here, B is the mini-batch, and *R* is the number of molecular features.
2. Bond-type head (edge-level). In each mini-batch, 15% of edges are randomly masked by zeroing their edge features. For each masked bond (*i*, *j*), the concatenated node embeddings [*h*_*i*_||*h*_*j*_] are input to a two-layer MLP to predict the 4-way bond class (single/double/triple/aromatic). The loss is cross-entropy (eq 4).
4 $$L_{\mathrm{bond}}=-\frac{1}{M}\sum_{e\in M}\log\frac{\exp\left(z_{e,y_{e}}\right)}{\sum_{c=1}^{4}\exp(z_{e,c})}$$
  where M is the set of masked edges in the mini-batch, $z_{e,c}\in R$ are the logits from the MLP for edge *e* and class *c* ∈ {1, ···,4} (single, double, triple, aromatic), and *y*_*e*_ is the ground-truth class index for edge *e*.
3. Node-reconstruction head (node-level). Fifteen percent of nodes are randomly masked by zeroing their node features, and a small MLP is trained to reconstruct the original features from the corresponding *h*_*i*_. The loss $L_{\mathrm{node}}$ is MSE identical to eq 3, applied to the masked-node feature vectors (i.e., replace *d̂* ^(*b*)^, *d* ^(*b*)^, with *x̂* ^(*b*)^ (the predicted node-feature vector), *x* ^(*b*)^ (the original node-feature vector); and let R → P, the number of node-features).
Equation 5 defines the total objective is a weighted sum:
5 $$L=\lambda_{\mathrm{desc}}L_{\mathrm{desc}}+\lambda_{\mathrm{bond}}L_{\mathrm{bond}}+\lambda_{\mathrm{node}}L_{\mathrm{node}}$$
  The weighting coefficients were set to (λ_desc_, λ_bond_, λ_node_) = (1.0, 0.5, 0.5). In Section 2.2, Pearson analyses indicate that molecular-level descriptors exhibit consistently stronger associations with transition temperature than atom- or bond-level features. Accordingly, the descriptor objective is prioritized to align pretraining with the most informative signals. λ_desc_ = 1.0 emphasizes molecule-level cues that correlate with transition temperature, while λ_bond_ = λ_node_ = 0.5 retain local chemical fidelity.

**Figure 3:**
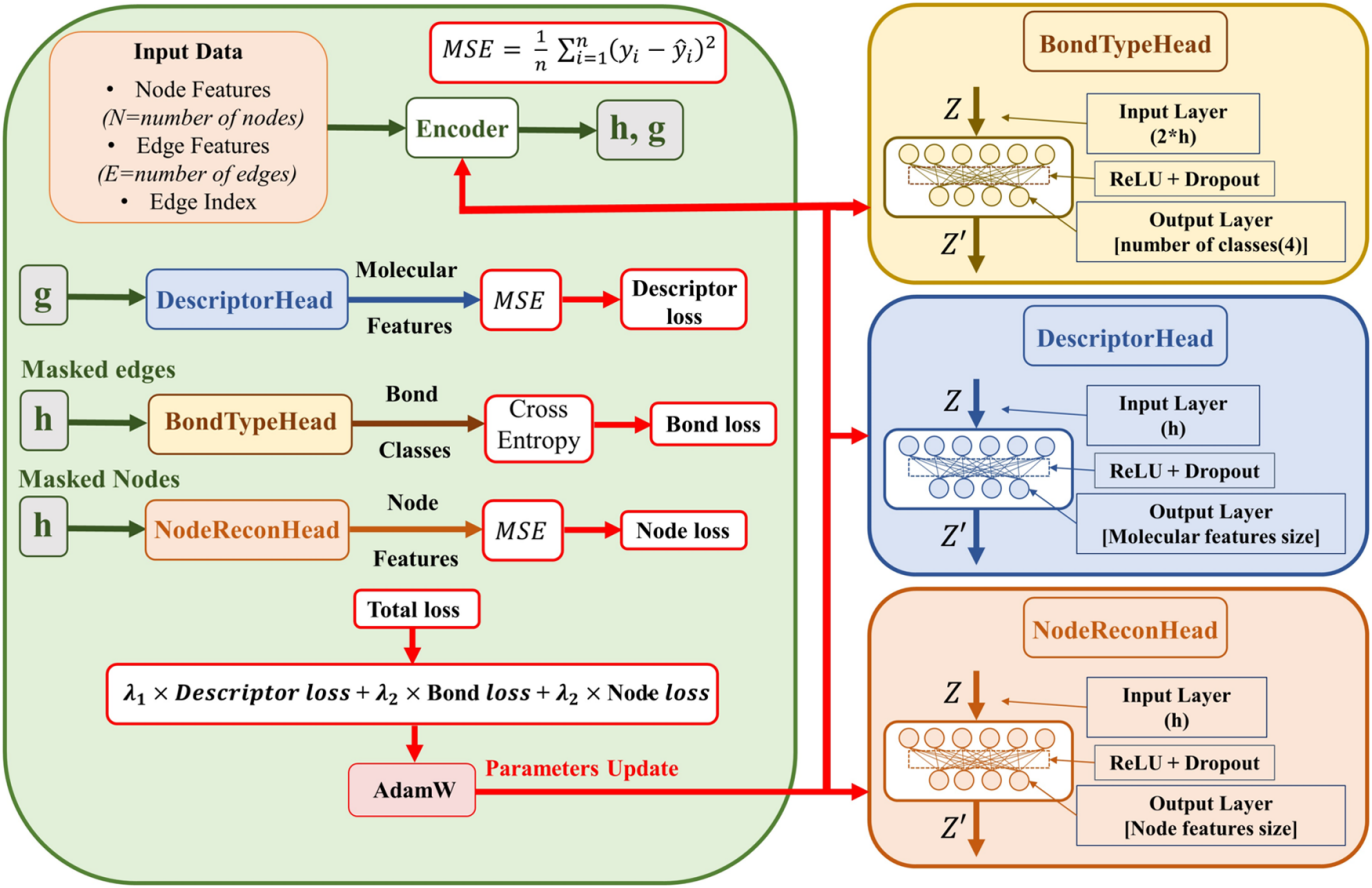
Overview of the PolyT-GNN pretraining model with three parallel heads attached to the pretrained GINE encoder. Given a polymer graph, the encoder outputs node embeddings *h* and a graph embedding *g*. *z* indicates the latent vector fed into each head.

To optimize PolyT-GNN’s performance, automated hyperparameter tuning was performed using the Optuna framework (Table S7). The encoder width was selected to be 64 based on a latent-size sweep. The full details of the hyperparameter search protocol—including search ranges, optimization strategy, and the selected final configuration—are provided in Section S2 of the Supporting Information.

During fine-tuning, the model is initialized with the pretrained PolyT-GNN encoder and adapted to the labeled 2W-SMP data set using a lightweight task head, while only partial encoder parameters are updated. Concretely, all encoder parameters are frozen except the last GINEConv block and its BatchNorm, and train them jointly with the task head using AdamW (learning rate 1 × 10^–5^, weight decay 1 × 10^–5^), StepLR scheduling (step size 250, γ = 0.1), and gradient-norm clipping (||∇|| ≤ 5); mini-batches of size 16 are used. To preserve the chemical priors learned in pretraining while adapting to the new task, the same masking strategy is retained during tuning: 15% of nodes and 15% of edges are randomly masked per batch to compute auxiliary node-reconstruction (MSE) and bond-type (cross-entropy) losses alongside the supervised regression loss on transition temperature. This joint optimization strategy serves as an effective regularization mechanism in the small-data regime and helps maintain informative graph representations, as demonstrated by Hu et al.,^76^ who showed that incorporating auxiliary self-supervised objectives during downstream training can enhance generalization and mitigate degradation of learned representations in graph neural networks.

After fine-tuning the encoder, a single mixture vector was formed for each monomer by summing monomer embeddings weighted by their experimental weight ratios and using these fingerprints as inputs to three non-neural regressors to predict transition temperature. The models trained include (i) a Support Vector Regressor (SVR) with a radial basis function (RBF) kernel, (ii) Gaussian Process Regression (GPR) with a Constant × RBF kernel plus a white-noise term, and (iii) Kernel Ridge Regression (KRR) with RBF. For each model, a scikit-learn pipeline was built, performed a 5-fold CV grid search over the key hyperparameters (Summary of the hyperparameters for each model is presented in Table S8), and refit on the best-MAE configuration. Data were split 85/15 into train/test with a fixed seed; labels were normalized using eq 2.

## 3 Results

### 3.1 Selection of Regression Model

Before assessing the effect of pretraining and fine-tuning, several regression algorithms were compared using the same pretrained latent vectors to identify the most suitable approach for mapping the latent representations to transition temperature (Table 1). Based on the latent dimension analysis presented in Table S5, the pretrained encoder with a latent dimension of 64 was selected for this comparison due to its superior generalization performance across all tested configurations as given in Table 1. Three widely used nonlinear regressors were evaluated: SVR, KRR, and GPR —using 5-fold cross-validation to ensure statistical reliability. Among them, the SVR model achieved the highest predictive accuracy with an average *R* ^2^ = 0.8293, outperforming both KRR (*R* ^2^ = 0.7745) and GPR (*R* ^2^ = 0.7944). The superior performance of SVR can be attributed to its ability to handle high-dimensional, nonlinear latent features using the radial basis function (RBF) kernel while maintaining robustness against overfitting in small data sets. Consequently, to maintain a consistent and controlled framework, the SVR model and latent dimension (64) were kept fixed in all subsequent analyses, and only the encoder representations were varied.

**Table 1:** Comparison of Different Regression Algorithms Applied to the Latent Vectors for Predicting the Transition Temperature of 2W-SMPs^a^

	Train			Test		
Models	*R* ^2^	MAE	MSE	*R* ^2^	MAE	MSE
SVR model	0.9607 ± 0.0028	0.0305 ± 0.0012	0.0015 ± 0.0001	**0.8293 ± 0.0447**	**0.0546 ± 0.0118**	**0.0069 ± 0.0040**
KRR model	0.9738 ± 0.0015	0.0209 ± 0.0014	0.0010 ± 0.0001	0.7745 ± 0.0853	0.0553 ± 0.0098	0.0087 ± 0.0029
GPR model	**0.9826 ± 0.0013**	**0.0169 ± 0.0008**	**0.0007 ± 0.0000**	0.7944 ± 0.1602	0.0518 ± 0.0217	0.0089 ± 0.0078

a Values are reported as mean ± standard deviation obtained from cross-validation.

### 3.2 Ablation Analysis

The ablation study summarized in Table 2 systematically evaluates the contribution of different input feature combinations to the prediction accuracy of transition temperatures. The results reveal that incorporating multiple feature types generally enhances model performance compared to using single feature groups alone. Among the partial combinations, the Node + Molecular configuration achieved the highest test *R* ^2^ of 0.7859, indicating a complementary relationship between atomic-level and global molecular descriptors. The Edge + Molecular and Node + Edge combinations showed comparable but slightly lower accuracies, suggesting that while bond connectivity contributes useful structural information, it is less dominant than atomic or molecular descriptors in isolation. Notably, the Full model, which integrates node, edge, and molecular features, achieved the best overall performance with a test *R* ^2^ = 0.8293 and the lowest mean absolute error (0.0546), confirming that a comprehensive representation combining all structural hierarchies yields the most accurate and generalizable predictions. These results, obtained through 5-fold cross-validation, demonstrate the synergistic importance of including both local (atomic and bonding) and global (molecular) information in the PolyT-GNN framework.

**Table 2:** Ablation Analysis of Input Feature Combinations for Transition Temperature Prediction^a^

	Train			Test		
Input features	*R* ^2^	MAE	MSE	*R* ^2^	MAE	MSE
Node + Molecular	0.9464 ± 0.0170	0.0183 ± 0.0025	0.0021 ± 0.0007	0.7859 ± 0.1136	0.0500 ± 0.0170	0.0092 ± 0.0078
Edge + Molecular	**0.9926 ± 0.0025**	**0.0060 ± 0.0009**	**0.0003 ± 0.0001**	0.7817 ± 0.0783	0.0538 ± 0.0093	0.0084 ± 0.0040
Node + Edge	0.9892 ± 0.0018	0.0082 ± 0.0008	0.0004 ± 0.0001	0.7566 ± 0.1028	0.0565 ± 0.0160	0.0098 ± 0.0062
Node	0.9520 ± 0.0129	0.0304 ± 0.0011	0.0019 ± 0.0005	0.6671 ± 0.2184	0.0671 ± 0.0133	0.0124 ± 0.0078
Molecular	0.8797 ± 0.0160	0.0353 ± 0.0027	0.0047 ± 0.0006	0.6689 ± 0.1571	0.0656 ± 0.0145	0.0127 ± 0.0067
Full	0.9607 ± 0.0028	0.0305 ± 0.0012	0.0015 ± 0.0001	**0.8293 ± 0.0447**	**0.0546 ± 0.0118**	**0.0069 ± 0.0040**

a Values are reported as mean ± standard deviation obtained from cross-validation.

### 3.3 Polymer Property Prediction Results

To evaluate the influence of pretraining and fine-tuning on prediction accuracy, three models were examined. The performance comparison presented in Table 3 underscores the critical role of pretraining and fine-tuning in enhancing the predictive capability of the PolyT-GNN framework. The direct model, trained exclusively on the limited 2W-SMP data set, achieved a high training accuracy (*R* ^2^ = 0.9732) but exhibited a pronounced decline in generalization performance (*R* ^2^ = 0.6092) on the test set. This disparity indicates a tendency toward overfitting, which is expected when training is conducted on a small and chemically heterogeneous data set without prior exposure to broader structural patterns. The direct model’s limited generalization emphasizes the need for knowledge transfer from large, diverse polymer corpora to capture chemically meaningful representations.

**Table 3:** Comparison of Model Performance for Predicting the Transition Temperature of 2W-SMPs^a^

	Train			Test		
Models	*R* ^2^	MAE	MSE	*R* ^2^	MAE	MSE
Direct model	**0.9732 ± 0.0038**	**0.0181 ± 0.0010**	**0.0010 ± 0.0000**	0.6092 ± 0.0033	0.0782 ± 0.0073	0.0152 ± 0.0019
Pretrained model	0.9607 ± 0.0028	0.0305 ± 0.0012	0.0015 ± 0.0001	0.8293 ± 0.0447	0.0546 ± 0.0118	0.0069 ± 0.0040
Finetuned model	0.9608 ± 0.0035	0.0303 ± 0.0010	0.0015 ± 0.0001	**0.8385 ± 0.0673**	**0.0529 ± 0.0146**	**0.0066 ± 0.0051**

a Values are reported as mean ± standard deviation obtained from cross-validation.

In contrast, both the pretrained and fine-tuned models demonstrated markedly improved predictive performance and generalization stability. The pretrained model, initialized using latent representations learned from the PI1M data set, achieved a test *R* ^2^ of 0.8293, representing a substantial improvement over the direct approach. Subsequent fine-tuning on the 2W-SMP data set further enhanced the predictive accuracy to *R* ^2^ = 0.8385, accompanied by the lowest mean absolute error (0.0529) and mean squared error (0.0066). The parity plot shown in Figure 4 illustrates the relationship between predicted and experimental transition temperatures for both the training (blue) and test (red) data sets. The training data exhibit a strong correlation with the experimental values, indicating that the model effectively captures the underlying structure–property relationships. Importantly, the test data also closely follow the ideal parity line, demonstrating good generalization performance despite a slightly increased dispersion compared to the training set. The corresponding training-fold parity plots provided in Figure S5 further demonstrate the model’s consistency across folds, with all exhibiting high *R* ^2^ values and low MAE and MSE. Collectively, these results validate the effectiveness of the transfer-learning strategy in polymer informatics, where pretraining captures generalized chemical and topological relationships, and fine-tuning enables adaptation to the specific characteristics of 2W-SMPs.

**Figure 4:**
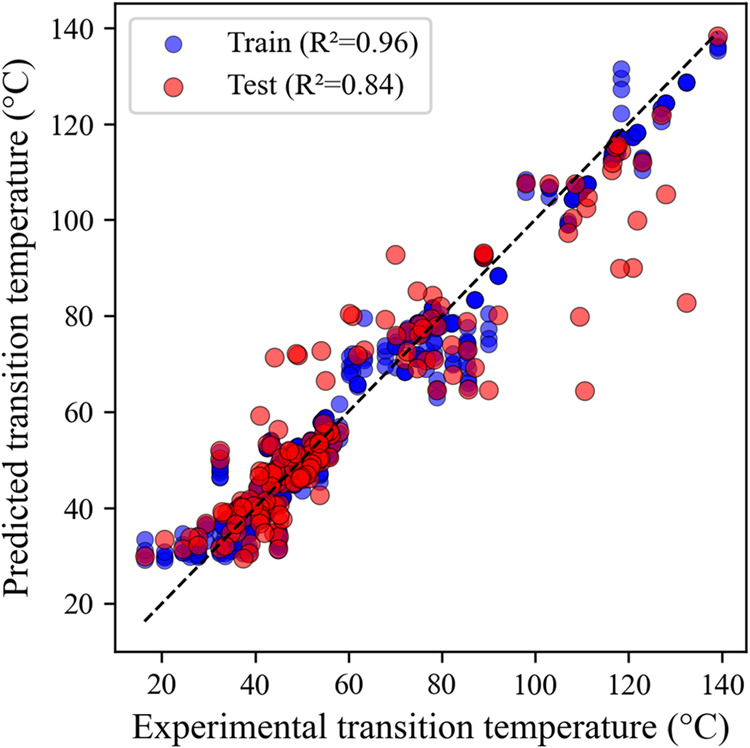
Parity plot of predicted versus experimental transition temperatures for both training (blue) and test (red) data sets. Training points are obtained from in-sample predictions across 5-fold cross-validation, while test points correspond to an independent hold-out set. The strong alignment of both data sets with the ideal diagonal line indicates high predictive accuracy and robust generalization of the model.

To further highlight the importance of self-supervised pretraining, a baseline study was conducted using only molecular descriptors of the constituent monomers, without leveraging any pretrained representations. In this case, the molecular features were first constructed as weighted molecular vectors following the same strategy illustrated in Figure 1, where the descriptor vectors of individual monomers are combined according to their corresponding weight ratios. These resulting mixture-level feature vectors were then directly used as input to conventional regression models (SVR, KRR, and GPR), and the results are summarized in Table 4. Despite achieving relatively strong training performance, all models exhibit significantly reduced generalization on the test set. The best test performance is obtained by the SVR model with an R^2^ of 0.6717, which is substantially lower than the best-performing model in Table 3 (R^2^ = 0.8385 for the fine-tuned PolyT-GNN). This corresponds to an improvement of approximately 25% in predictive accuracy when leveraging pretrained and fine-tuned graph-based representations. The noticeable performance gap demonstrates that relying solely on handcrafted molecular descriptors is insufficient to capture the complex structure–composition–property relationships governing 2W-SMPs. In contrast, the self-supervised pretraining strategy enables the model to learn richer and more transferable chemical representations from large-scale polymer data, which significantly enhances generalization performance in the limited-data regime.

**Table 4:** Performance Comparison of SVR, KRR, and GPR Models for Predicting Transition Temperature Using Only Molecular Descriptors of Constituent Monomers, without Pretraining^a^

	Train			Test		
Models	*R* ^2^	MAE	MSE	*R* ^2^	MAE	MSE
SVR model	0.8901 ± 0.0271	0.0237 ± 0.0029	0.0044 ± 0.0013	**0.6717 ± 0.1752**	**0.0665 ± 0.0247**	**0.0147 ± 0.0125**
KRR model	0.8853 ± 0.0152	0.0408 ± 0.0039	0.0045 ± 0.0009	0.5636 ± 0.2418	0.0783 ± 0.0227	0.0185 ± 0.0142
GPR model	**0.9654 ± 0.0115**	**0.0215 ± 0.0043**	**0.0013 ± 0.0004**	0.3873 ± 0.3568	0.0837 ± 0.0289	0.0252 ± 0.0185

a Results are reported as mean ± standard deviation over five-fold cross-validation.

### 3.4 Latent Space Evaluation

To assess how fine-tuning reshapes the polymer representation space, the latent vectors of the PI1M and 2W-SMP data sets were visualized using t-distributed stochastic neighbor embedding (t-SNE) (Figure 5). Prior to fine-tuning, the 2W-SMP monomers (red) are broadly dispersed throughout the PI1M polymer manifold (gray), exhibiting a largely isotropic distribution with no clear structural organization, which suggests limited alignment with task-specific features. In contrast, after fine-tuning, the 2W-SMP embeddings exhibit a more structured and anisotropic distribution, with reduced randomness and improved local coherence. Notably, a clear directional shift of the embeddings toward a specific region of the latent space is observed, indicating that the model systematically reorganizes the representation of SMP monomers in response to domain-specific information. This redistribution suggests that chemically relevant similarities are better captured after fine-tuning. Importantly, this refinement occurs without disrupting the global structure of the pretrained PI1M space, demonstrating that fine-tuning enhances domain-specific representation while preserving the underlying transferable chemical knowledge.

**Figure 5:**
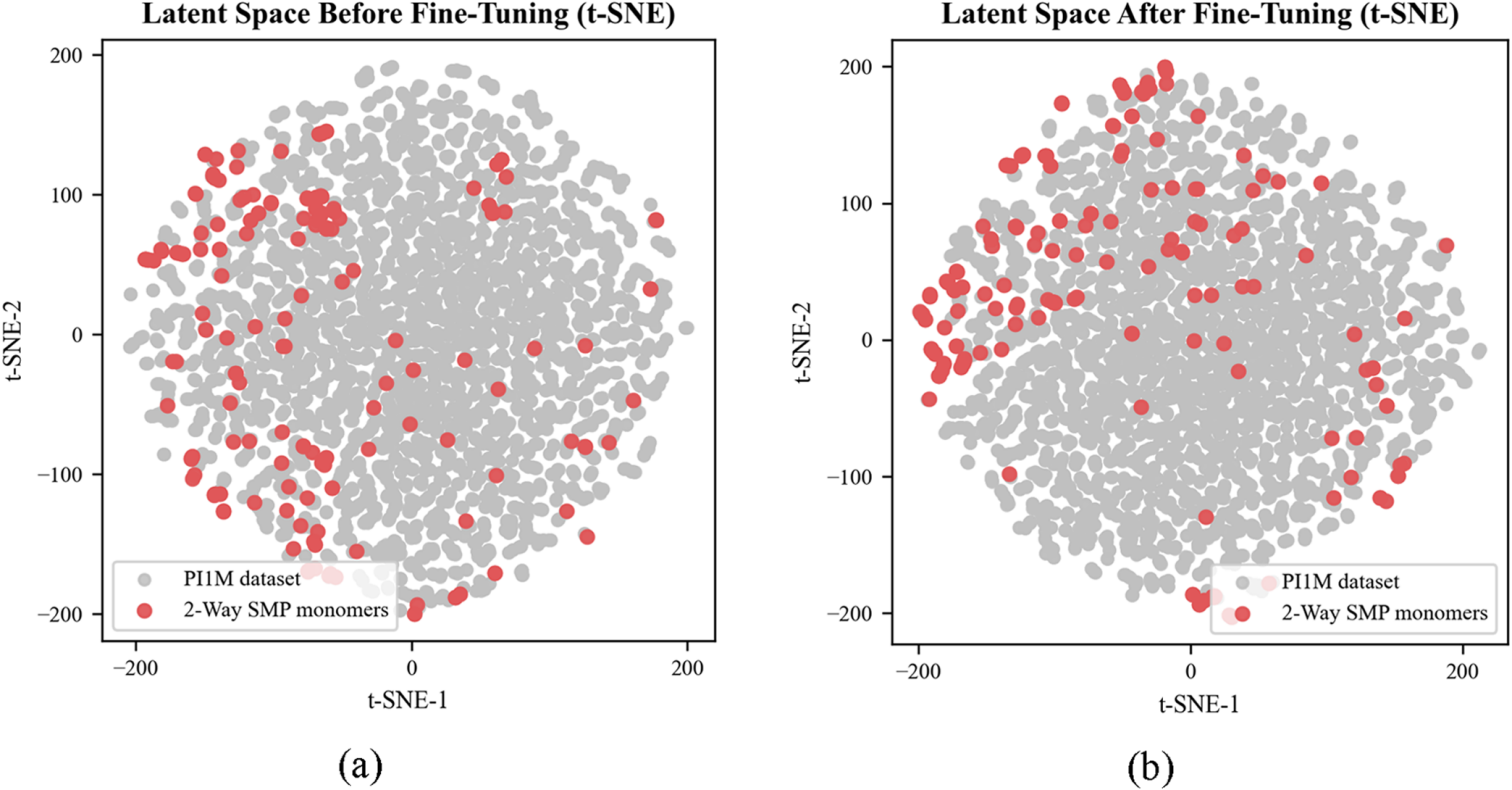
t-SNE visualization of the latent space before (a) and after (b) fine-tuning. Red points represent 2W-SMP monomers and gray points denote the PI1M polymer data set.

To quantify these changes, two complementary metrics described in the Supporting Information—namely the Silhouette coefficient (Section S3) and the Maximum Mean Discrepancy (MMD) (Section S4)—were computed. The Silhouette coefficient measures the compactness and separation of clusters within the latent space, whereas the MMD evaluates the statistical distance between the distributions of 2W-SMP and general polymer embeddings.

As summarized in Table 5, the Silhouette coefficient increased from 0.413 to 0.483 (Δ = +0.071) following fine-tuning, indicating stronger intracluster cohesion and clearer intercluster boundaries among the 2W-SMP representations. Concurrently, the MMD decreased slightly from 0.0920 to 0.0895 (Δ = −0.0024), reflecting a modest improvement in local alignment between the fine-tuned 2W-SMP and PI1M manifolds. Together, these quantitative metrics confirm that fine-tuning enhances the structural organization and chemical interpretability of the latent space, producing more distinct and physically meaningful embeddings for 2W-SMPs while maintaining continuity with the broader polymer feature distribution.

**Table 5:** Unsupervised Cluster Quality and Distribution Distance of the 2W-SMP Latent Space before and after Fine-Tuning^a^

Metric	Before fine-tuning	After fine-tuning	Δ (Change)
Silhouette	0.413	0.483	+0.071
MMD	0.0920	0.0895	–0.0024

a Values are reported as mean ± standard deviation obtained from cross-validation.

### 3.5 Cross-Domain Generalization to One-Way SMPs

To assess the transferability of the pretrained representation, the PolyT-GNN was evaluated on a separate data set of one-way SMPs (1W-SMPs), where the Tg governs actuation. The pretrained model used the encoder trained on the PI1M data set without adaptation, while the fine-tuned model employed the same encoder further trained on the 1W-SMP data set previously introduced by our research group in a recent publication.^35^ The latent vectors of the PI1M and 1W-SMP data sets were visualized using t-SNE in Figure S6. As shown in Table 6, both models achieved strong predictive accuracy, with test *R* ^2^ values of 0.8058 and 0.8133, respectively. Fine-tuning slightly improved performance and reduced error (MAE = 0.0638 vs 0.0727), confirming that the pretrained encoder captures transferable polymer features while adapting effectively to domain-specific SMP data.

**Table 6:** Performance of the Pretrained and Fine-Tuned PolyT-GNN Models (Fine-Tuned on One-Way SMPs) in Predicting the *T*_g_ of One-Way SMPs^a^

	Train			Test		
Models	*R* ^2^	MAE	MSE	*R* ^2^	MAE	MSE
Pretrained model	0.9146 ± 0.0057	0.0496 ± 0.0032	0.0045 ± 0.0008	0.8058 ± 0.0242	0.0727 ± 0.0083	0.0103 ± 0.0024
Finetuned model	**0.9707 ± 0.0020**	**0.0309 ± 0.0009**	**0.0015 ± 0.0001**	**0.8133 ± 0.0500**	**0.0638 ± 0.0112**	**0.0100 ± 0.0043**

a Values are reported as mean ± standard deviation obtained from cross-validation.

The parity plot in Figure S7 illustrates the correlation between predicted and experimental transition temperatures for the 1W-SMP data set, demonstrating the model’s strong predictive capability across 5-fold cross-validation. The corresponding training-fold parity plots in Figure S8 further confirm the stability and reproducibility of the model, with all folds exhibiting high *R* ^2^ and low MAE and MSE. Quantitative analysis of cluster quality and distribution alignment is summarized in Table S9, both illustrating the improved clustering and organization of 1W-SMPs embeddings following fine-tuning.

### 3.6 Comparison with Related Studies

To benchmark the predictive performance of the proposed framework, the results were compared with recent polymer informatics studies that employed data sets of comparable size (Table 7). Despite utilizing only 170 data points, the present GAE + SVM model achieved an *R* ^2^ of 0.84, surpassing models such as XGBoost (*R* ^2^ = 0.65),^77^ DNN (*R* ^2^ = 0.66),^78^ and SVM (*R* ^2^ = 0.69),^79^ and performing on par with transformer-based and LSTM architectures trained on larger data sets.^79,80^

**Table 7:** Comparison of the Proposed GAE + SVM Model with Recent Polymer Informatics Studies Using Limited Datasets

Study	Method/Model	Data set size	Predicted property	*R* ^2^
**PolyT-GNN**	**GAE + SVM**	**170**	**Transition temperature**	**0.84**
Malashin et al.^77^	XGBoost	176	Oxygen index	0.65
Shi et al.^78^	DNN	200	Polymer–Surface	0.66
			Adhesion Strength	
Xu et al.^81^	Transformer-based language model	271	Conductivity	0.73
Fatriansyah et al.^79^	SVM	1437	Glass Transition Temperature	0.69
Ahn et al.^80^	LSTM (Topo-HAPPY)	456	Solubility	0.615

This comparison highlights the efficiency of the proposed graph-based latent representations combined with SVM regression in achieving high accuracy under limited-data conditions, underscoring the framework’s suitability for small, experimentally derived polymer data sets.

### 3.7 High-Throughput Screening of Candidate 2W-SMPs

To identify promising two-way shape memory polymers (2W-SMPs) with elevated transition temperatures, a high-throughput screening strategy was employed based on systematic enumeration of monomer combinations. All monomers and cross-linkers in the curated data set were paired in binary combinations, and for each pair, the composition was varied by adjusting their weight ratio from 5/95 to 95/5 in increments of 5%. This resulted in 19 distinct compositions per monomer pair, covering a broad compositional space.

To ensure a fair evaluation and prevent data leakage, formulations already present in the original 2W-SMP data set were excluded from the screening set. Following this filtering step, the final design space encompassed 80693 unique 2W-SMP formulations, each defined by its constituent monomers and the specific weight ratio between them. Similar to the mentioned process above the graph representation of the monomer pairs were created. PolyT-GNN was used to predict the transition temperature of all candidate formulations. Due to the desired application of the formulated 2W-SMPs in high temperature environments, the goal is to identify polymers having transition temperature above 100 °C. Table 8 presented three predicted 2W-SMPs using the PolyT-GNN framework. The newly designed 2W-SMPs were screened primarily based on their predicted transition temperatures obtained from PolyT-GNN. We also considered their potential to form semicrystalline structures. Formulations with transition temperatures above 100 °C were prioritized, as elevated transition temperatures are a key requirement for achieving stable thermally driven reversible actuation in high-temperature environments.

**Table 8:**
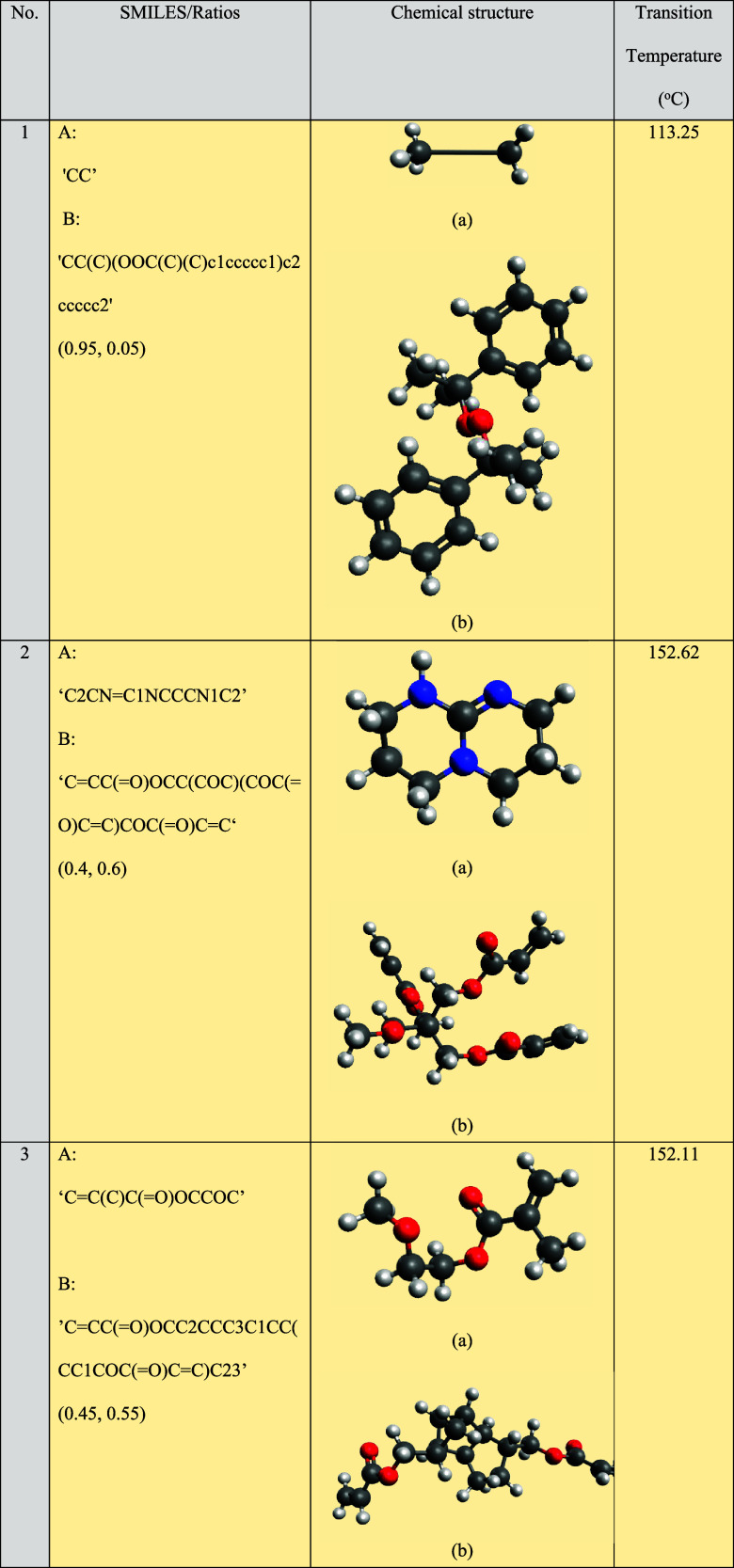
Candidate 2W-SMP Formulations Selected for Experimental Validation, Including Monomer SMILES and Weight Ratios, Molecular Structures, and Predicted Transition Temperatures^a^

a Color scheme: red = Oxygen, black = Carbon, silver = Hydrogen, light blue = Silicon, blue = Nitrogen.

The first candidate was selected as a proof-of-concept material based on its favorable processability, commercial availability of its constituents, and well-established reaction chemistry, which collectively enable reliable and reproducible experimental validation of the proposed framework. Importantly, this formulation was not included in the training data set and was intentionally selected as a well-understood system to rigorously evaluate the model’s ability to generalize to unseen data. Although its predicted transition temperature (∼130 °C) does not reach the extreme conditions required for some applications such as in geothermal systems, it still represents relatively high-temperature actuation compared to most reported 2W-SMPs, which typically exhibit transition temperatures below 100 °C (Figure S1). In contrast, the other two candidates correspond to the highest predicted transition temperatures within the explored design space, representing the upper-performance limit identified based on the available training data set and constituting a meaningful step toward the long-term objective of developing 2W-SMPs capable of operating near or above 200 °C. To the best of our knowledge, these formulations have not been previously reported in the literature, highlighting the capability of the framework to propose genuinely novel material systems. However, due to the increased synthetic complexity of these candidates and the scope of the present study, their experimental validation was not pursued. Once these predicted new 2W-SMPs are validated experimentally in the near future either by our lab or by other research laboratories, they can be added to our training data set, which will facilitate prediction of 2W-SMPs with even higher transition temperatures, gradually achieving the long-term goals. These predictions are expected to provide valuable guidance for future experimental investigations in polymer chemistry aimed at realizing next-generation high-temperature 2W-SMPs.

### 3.8 Synthesis and Characterization

The selected monomers are polyethylene (PE) and dicumyl peroxide (DCP). Polyethylene (Aldrich, Cat. No. 429015, purity ≥ 99%) and dicumyl peroxide (98%, Aldrich, Cat. No. 329541) were used as received without further purification. Toluene (Sigma-Aldrich, Cat. No. 244511) and chloroform (Sigma-Aldrich, Cat. No. 288306) served as solvents. To prepare the PE–DCP system, 100 g of PE (95 wt %) was dissolved in 100 mL Xylene at 100 °C under continuous stirring for 24 h. In parallel, 5.26 g of DCP (5 wt %) was dissolved in 70 mL chloroform at room temperature for 30 min to ensure complete dispersion. The two solutions were then combined and mixed at 100 °C for 1 h to obtain a homogeneous polymer–cross-linker mixture. Given the high thermal sensitivity of the resulting solution, particular care was taken during solvent removal. Instead of glass containers, disposable paper cups were employed, as they minimized localized overheating and facilitated handling at elevated temperature. Each paper cup was first preheated in a conventional oven until its temperature stabilized at 100 °C. This step prevented thermal shock and ensured that the subsequently introduced polymer solution remained at a uniform temperature. Precisely 20 mL of the hot polymer solution (maintained at 100 °C) was poured into each preheated cup, which was then immediately transferred to a vacuum oven set at 100 °C. Solvent removal was carried out under a reduced pressure of 0.08 MPa for 12 h, allowing gradual evaporation of residual Xylene and chloroform without inducing premature cross-linking or degradation of the mixture. Afterward, the samples were thermally cured in an oven at 100 °C for 30 min. At this temperature, DCP undergoes sufficient thermal activation to generate radicals, enabling the onset of cross-linking within the polyethylene matrix.^82^ This curing step facilitated cross-linking and the formation of a polymer network.^83,84^ Final samples have been studied using Dynamic Mechanical Analysis (DMA) and Differential Scanning Calorimetry (DSC).

Figure 6 shows the DSC thermogram of the synthesized polymer, in which the heat flow is plotted as a function of temperature. Thermal analysis was carried out using a PerkinElmer DSC 4000 under a continuous nitrogen purge (20.0 mL·min^–1^) to prevent oxidative degradation. Approximately 7.90 mg of the polymer sample was sealed in an aluminum pan. The instrument was equilibrated at 25 °C for 3 min, followed by heating from 25 to 180 °C at a rate of 10 °C·min^–1^. The sample was then held isothermally at 180 °C for 3 min before being cooled back to 25 °*C* at 10 °C·min^–1^. A final isothermal segment of 3 min at 25 °C completed the program. Heat flow (mW) was continuously recorded as a function of temperature throughout the heating–cooling cycle. To remove the influence of thermal history associated with curing and processing, the DSC measurement was conducted twice. The thermal properties reported here correspond to the second heating/cooling cycle, which reflects the intrinsic transitions of the PE–DCP system.

**Figure 6:**
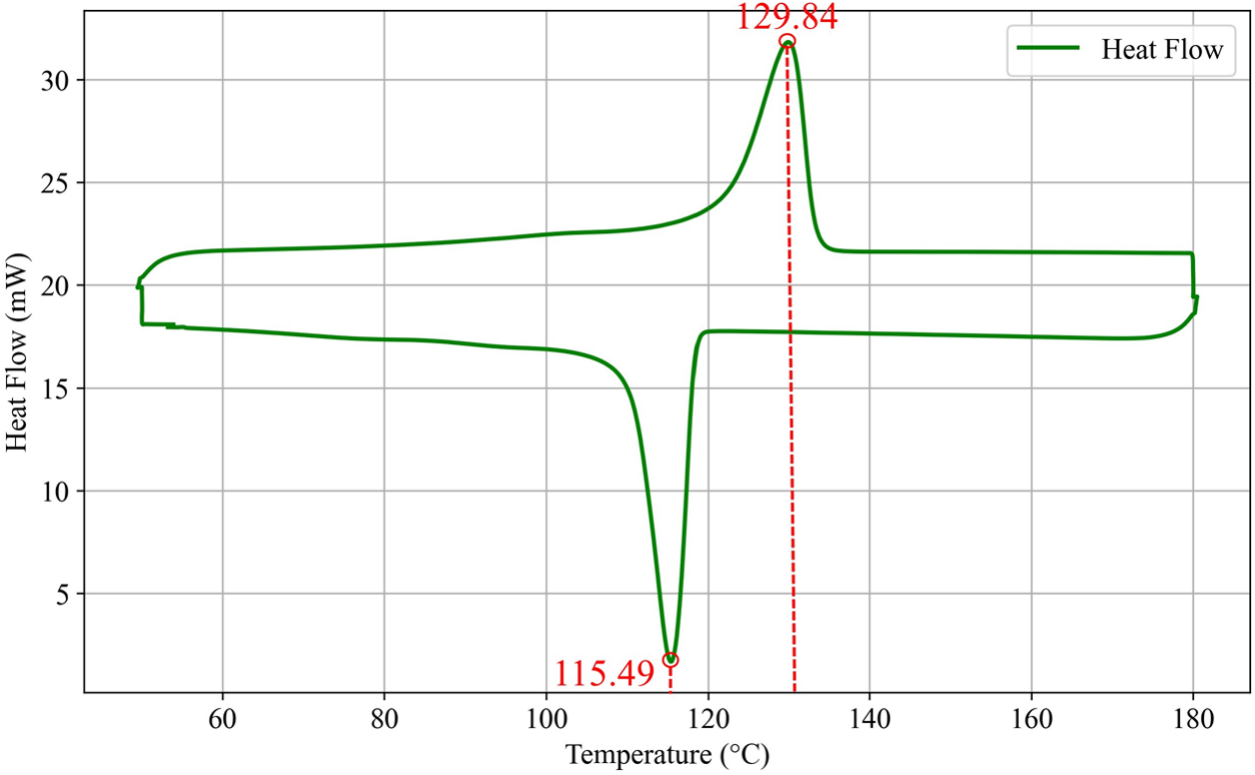
DSC thermogram (second heating/cooling cycle) showing melting peak near 130 °C and crystallization temperature about 115 °C, confirming the polymer’s semicrystalline structure.

The thermogram exhibits a distinct endothermic shift followed by an exothermic peak, which are characteristic of the polymer’s thermal transitions. DSC analysis of the selected sample revealed a sharp endothermic peak at approximately 129.84 °C, which is indicative of a melting transition rather than a glass transition. Such a distinct melting behavior confirms that the system exhibits semicrystalline characteristics. The presence of semicrystalline domains in this SMP provides the necessary reversible phase transition mechanism for reversible actuation, where crystallization upon cooling induces elongation and melting upon heating drives contraction. These results therefore verify that the synthesized material is a semicrystalline 2W-SMP, consistent with the molecular composition containing polyethylene-based segments capable of forming crystalline phases.

The transition temperature of the selected semicrystalline 2W-SMP, determined from the DSC thermogram, was approximately 129.84 °C. In comparison, the PolyT-GNN model predicted a melting transition temperature of 113.25 °C for the same formulation. The deviation between experimental and predicted values corresponds to an error of about 12.78%, which is well within an acceptable margin considering the complexities of polymer synthesis and thermal analysis. This close agreement underscores the capability of the PolyT-GNN framework to accurately predict the thermal switching behavior of 2W-SMPs and highlights its potential as a reliable tool for guiding experimental discovery of new high-performance formulations.

Figure 7 presents thermomechanical behavior of the synthesized polymer which was characterized using a DMA in controlled-force mode. In this study, a cold-programming protocol was employed, in which the tensile bias was introduced at a temperature below the melting temperature (Tm). In semicrystalline networks, the crystalline domains act as netpoints while the amorphous phase serves as the switching segment; the applied stress induces internal bias that enables reversible actuation through stress-assisted crystallization during cooling and melting-induced contraction during heating.^29^ Compared to conventional hot programming (above Tm), cold programming generally produces a lower actuation strain but can generate higher programming stress and recovery stress. This approach is well suited for evaluating reversible actuation behavior under controlled mechanical bias conditions and is compatible with DMA-based thermomechanical testing.

**Figure 7:**
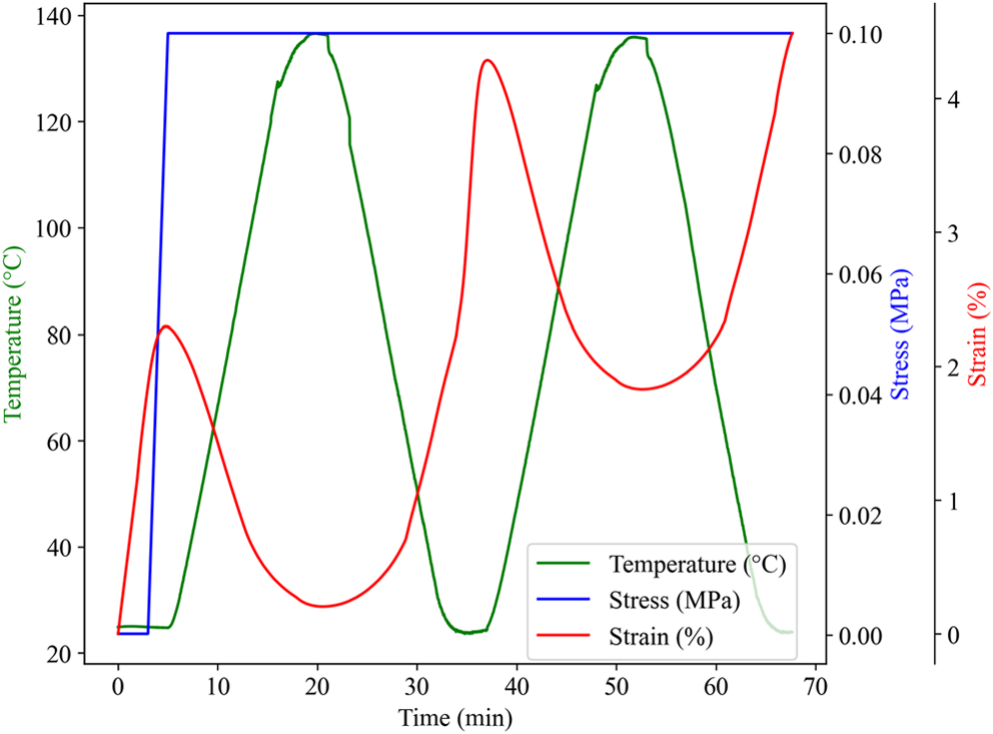
DMA results show reversible strain response during thermal cycling, confirming the two-way shape memory behavior of the polymer.

Rectangular film specimens with dimensions of approximately 9.72 mm × 4.60 mm × 2.15 mm (length × width × thickness) were clamped in tension configuration. A small preload force of 0.001 N was applied to ensure proper alignment. The thermal program began with equilibration at 25 °C followed by a 3 min isothermal hold. A constant stress ramp from 0.05 MPa·min^–1^ up to 0.10 MPa was then applied. The sample was subsequently heated from 25 to 135 °C at a rate of 10 °C·min^–1^ and held isothermally for 5 min. Cooling was performed from 135 to 25 °C at the same rate (10 °C·min^–1^), followed by another isothermal segment of 5 min. This heating–cooling cycle was repeated two times to assess reversible actuation under cyclic thermal loading. During the test, the evolution of stress, strain, and temperature was continuously recorded.

The curves show the evolution of temperature (green, left axis), stress (blue, middle axis), and strain (red, right axis) as functions of time. When the temperature increases from ∼25 to 135 °C, the strain gradually decreases, indicating contraction upon heating. Upon cooling back to 25 °C, the strain increases again, reflecting elongation. This reversible contraction–elongation behavior upon thermal cycling is characteristic of an intrinsic two-way shape memory response. Importantly, the stress remains nearly constant throughout the cycles, confirming that the actuation occurs under a constant external load. The repeated strain oscillations across multiple heating–cooling cycles validate the stable and reversible actuation capability of the polymer, underscoring its potential as a 2W-SMP suitable for high-temperature applications.

In Table 9, the actuation magnitude of 4.07% and recovery magnitude of 2.45% were extracted from the cooling and heating branches, respectively. Although the actuation strain may appear relatively small, such values are typical for cold-programmed two-way SMPs under constrained DMA conditions, where the reversible deformation is governed by stress-assisted crystallization and limited by the applied bias force.^85^ The consistent and reversible strain evolution during thermal cycling confirms the presence of a stable two-way shape memory effect.

**Table 9:** Quantitative Assessment of Actuation and Recovery Magnitudes Derived from the Strain-Temperature–Time Profiles in Figure 7

Phase	Time range	Strain change	Magnitude
Cooling	20–37 min	0.21% to 4.28%	Actuation 4.07%
Heating	37–52 min	4.28% to 1.83%	Recovery 2.45%

While the synthesized PE–DCP system successfully demonstrates a reversible two-way shape memory response, the actuation magnitude (∼4.07%) and recovery magnitude (∼2.45%) observed under the cold-programming DMA protocol are relatively modest. These values are characteristic of constrained, stress-assisted crystallization in semicrystalline networks tested under cold-programming conditions with a fixed bias force, and are not unexpected for this class of materials and testing configuration. Nevertheless, they highlight an important limitation of the current validation effort: the experimental campaign is primarily designed to confirm the predicted transition temperature rather than to optimize or fully characterize the actuation performance of the identified formulation. Achieving higher actuation strains and more complete shape recovery would likely require systematic tuning of cross-link density, programming stress, and thermal cycling parameters, which lie beyond the scope of the present study. Furthermore, the PolyT-GNN framework currently targets transition temperature as the primary design criterion, and does not explicitly incorporate microstructural or mechanical descriptors—such as crystallinity, cross-link density, or actuation strain—that more directly govern macroscopic two-way shape memory performance. Future extensions of this framework should therefore integrate such actuation-relevant properties as additional prediction targets, enabling a more complete structure–processing–property mapping and ultimately supporting the rational design of 2W-SMPs with both thermally appropriate switching temperatures and practically useful actuation amplitudes.

## 4 Conclusions

This study establishes PolyT-GNN as a data-efficient and transferable framework for the discovery of high-temperature two-way shape memory polymers (2W-SMPs) using graph neural networks (GNN). By integrating atomic, bond, and molecular descriptors with explicit monomer weight ratios, PolyT-GNN effectively bridges molecular structure, composition, and actuation behavior. Leveraging large-scale pretraining on the PI1M corpus and fine-tuning on a curated data set of 170 2W-SMPs, the model achieves high predictive accuracy (test *R* ^2^ = 0.84) despite limited data. Beyond 2W-SMPs, PolyT-GNN successfully generalizes to 1W-SMPs, accurately predicting their glass transition temperatures and confirming strong cross-domain transferability. Generative screening of over 80693 formulations further demonstrates its capacity for materials discovery, leading to the synthesis and experimental validation of a polyethylene–dicumyl-peroxide system with a melting transition near 130 °C, in close agreement with the predicted 113.25 °C. These results underscore PolyT-GNN’s potential as a robust and generalizable tool for accelerating the rational design of thermally stable SMPs and advancing data-driven polymer informatics toward next-generation smart materials.

## Supplementary Material



## Data Availability

The data that support the findings of this study are available from the corresponding author upon reasonable request.

## Notes

Detailed descriptions of the data set construction and quality-control criteria for two-way shape memory polymers; distributions of transition temperatures; correlation analyses of atomic and molecular descriptors; comprehensive lists and definitions of RDKit-derived atomic, bond-level, and molecular features; electronegativity values and bond polarity calculations; feature construction schematics; hyperparameter optimization details; and performance comparisons across latent dimensions and baseline machine-learning models; additional figures present parity plots, latent-space visualizations, and unsupervised clustering analyses, while tables summarize model configurations, descriptor definitions, and quantitative evaluation metrics (PDF)
