# Supplementary material for: PolyT-GNN: A Graph Neural Network Framework for Data-Driven Discovery of High-Temperature Two-Way Shape Memory Polymers
Source: ACS Appl Mater Interfaces. 2026 May 21;18(21):30373–88. doi: 10.1021/acsami.6c02689 (PMC13244374; doi:10.1021/acsami.6c02689)
Supplement: Supplementary file 1 [file am6c02689_si_001.pdf]

Supporting Information

PolyT-GNN: A Graph Neural Network Framework for Data-Driven Discovery of High-Temperature Two-Way Shape Memory Polymers

Amir Teimouri, Xiaowei Mu, Guoqiang Li*

Department of Mechanical & Industrial Engineering, Louisiana State University,

Baton Rouge, LA 70803, United States

*Corresponding author E-mail: lguoqi1@lsu.edu

Table of contents

Supplementary Figures:
**Figure S1.** Distribution of 2W-SMPs in the dataset across different transition temperature ranges. The majority of SMPs exhibit transition temperatures between 40 °C and 60 °C, indicating that most reported 2W-SMPs operate within this moderate thermal activation range.
**Figure S2.** Pearson correlation coefficients among the fourteen atomic features. The values within the matrix represent the correlation strength, where +1 and −1 correspond to perfectly positive and perfectly negative linear relationships, respectively.
**Figure S3.** Correlation matrix of the selected molecular descriptors with the transition temperature of the polymers (All molecular-level features and their descriptions are summarized in Table S3).
**Figure S4.** Feature construction for a representative monomer (SMILES: "CC") using RDKit. Atomic features (green) encode local properties such as aromaticity, ring membership, partial

charge, and number of implicit hydrogens. Bond-level edge features (orange) capture bond type, aromaticity, conjugation state, and electronegativity difference, with connectivity represented by the edge index (yellow). Global molecular descriptors (blue), including PEOE_VSA2, VSA_EState8, BCUT2D_MWLOW, and functional group counts, provide mixture-level information. This hierarchical representation integrates atom-, bond-, and molecule-level attributes as input to the graph neural network.
**Figure S5.** Parity plots of predicted versus experimental transition temperatures for the training data across five-fold cross-validation. Each panel corresponds to one training fold, demonstrating the strong agreement between predicted and experimental values, with all folds exhibiting high R^2 and low MAE and MSE.
**Figure S6.** t-SNE visualization of the latent space before (a) and after (b) fine-tuning on the one-way SMP dataset. Gray points represent polymers from the Pi1M dataset, and green points denote 1W-SMP monomers.
**Figure S7.** Parity plot of predicted versus experimental transition temperatures obtained from out-of-fold predictions across five-fold cross-validation. Each data point represents a 1W-SMP whose transition temperature was predicted by a model not trained on that sample, demonstrating strong agreement between predicted and experimental values.
**Figure S8.** Parity plots of predicted versus experimental transition temperatures for the one-way SMP dataset across five-fold cross-validation. Each panel corresponds to one training fold, demonstrating the strong agreement between predicted and experimental values, with all folds exhibiting high coefficients of determination (R^2) and low prediction errors (MAE and MSE).

Supplementary Tables:
**Table S1.** Criteria for Data Extraction and Quality Control of 2W-SMPs.
**Table S2.** Atomic features extracted from RDKit and their descriptions.
**Table S3.** Molecular descriptors extracted from RDKit and their descriptions.
**Table S4.** Pauling electronegativity values used for calculating bond polarity features.
**Table S5** Performance of the SVR model using latent vectors of different dimensions as input features.
**Table S6.** Effect of virtual-node embeddings on SVR prediction accuracy for polymer transition temperatures.
**Table S7.** Hyperparameter search space and selected configuration.
**Table S8.** Summary of the hyperparameter settings used for the baseline machine learning models in predicting the transition temperatures of 2W-SMPs.
**Table S9.** Unsupervised cluster quality and distribution distance of the 1W-SMP latent space before and after fine-tuning.

S1. Dataset development

Table S1. Criteria for Data Extraction and Quality Control of 2W-SMPs.

Criterion	Description
**Monomers and crosslinkers weight ratio reported**	Ensures formulation is fully defined and comparable across studies. Data was excluded if the weight ratio was missing or ambiguous.
**Experimental data contains reasonable trends and errors**	Confirms that reported transition temperatures follow expected trends and include measurement details. Data with unrealistic values or missing error margins were excluded.

**No significant concerns regarding sample preparation**	Verifies that synthesis/processing details were adequate (e.g., curing, crosslinking, thermal history). Publications with unclear or problematic sample preparation were excluded.
**Transition temperature measurement method specified**	Only data with clear indication of DSC, DMA, or optical analysis method were included, ensuring reproducibility.
**Actuation mechanism clearly identified**	Confirms that the selected transition temperature (T_m, T_g, or T_{ni}) corresponds to the switching domain driving the two-way actuation.

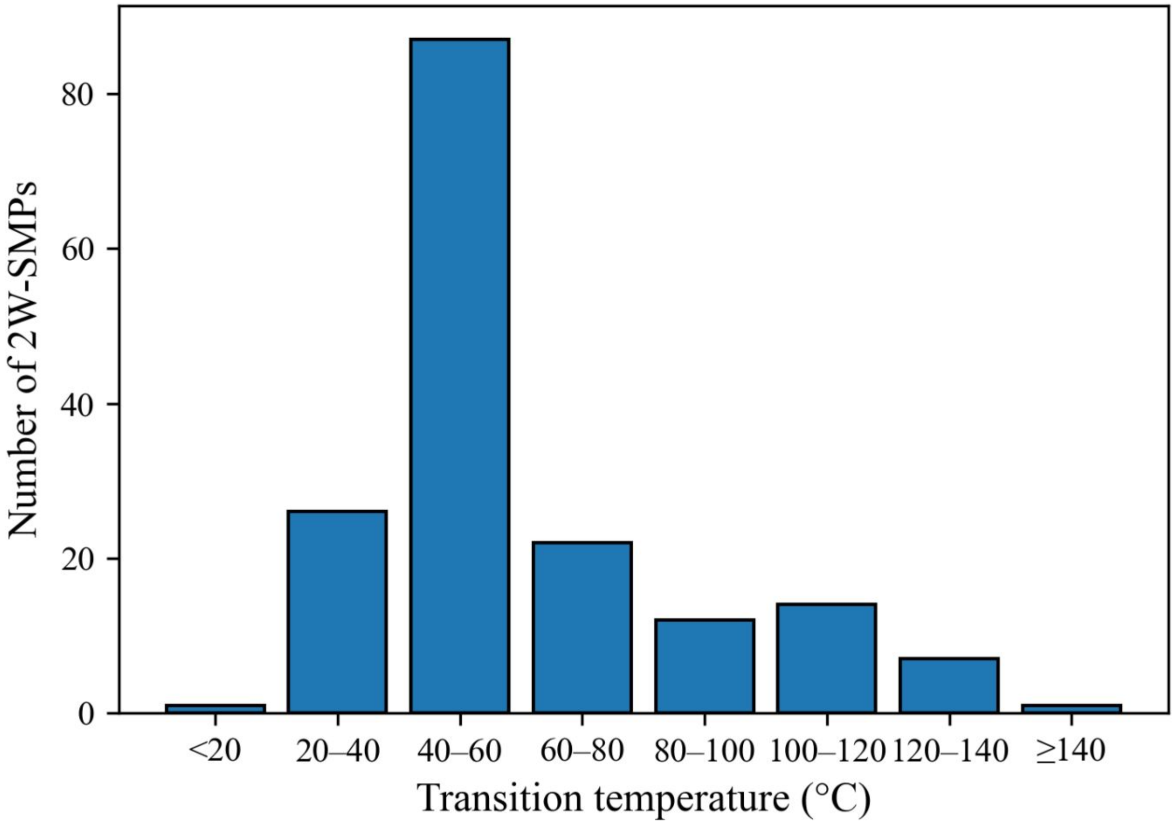

Figure S1. Distribution of 2W-SMPs in the dataset across different transition temperature ranges. The majority of SMPs exhibit transition temperatures between 40 °C and 60 °C, indicating that most reported 2W-SMPs operate within this moderate thermal activation range.

Table S2. Atomic features extracted from RDKit and their descriptions.

Feature	Description
**Atomic number**	Unique identifier of the chemical element.
**Total number of hydrogens**	Total number of hydrogen atoms attached to the atom.
**Degree**	Number of directly bonded neighboring atoms.
**Implicit valence**	Number of bonds expected by valence rules but not explicitly drawn, typically corresponding to implicit hydrogens.
**Aromaticity**	Indicates whether the atom is part of an aromatic system.
**Hybridization**	Hybridization state of the atom (e.g., sp, sp2, sp3).
**Atomic mass**	Standard atomic weight of the atom.
**Formal charge**	Net charge assigned to the atom.
**Ring membership**	Indicates if the atom is part of a ring structure.
**Radical electrons**	Number of unpaired (radical) electrons on the atom.
**Total valence**	Total valence considering explicit and implicit valences.
**No implicit flag**	Indicates whether implicit hydrogens are allowed for this atom.
**Explicit hydrogens**	Number of explicit hydrogen atoms attached to the atom.

**Implicit hydrogens**	Number of implicit hydrogen atoms assumed to be bonded.

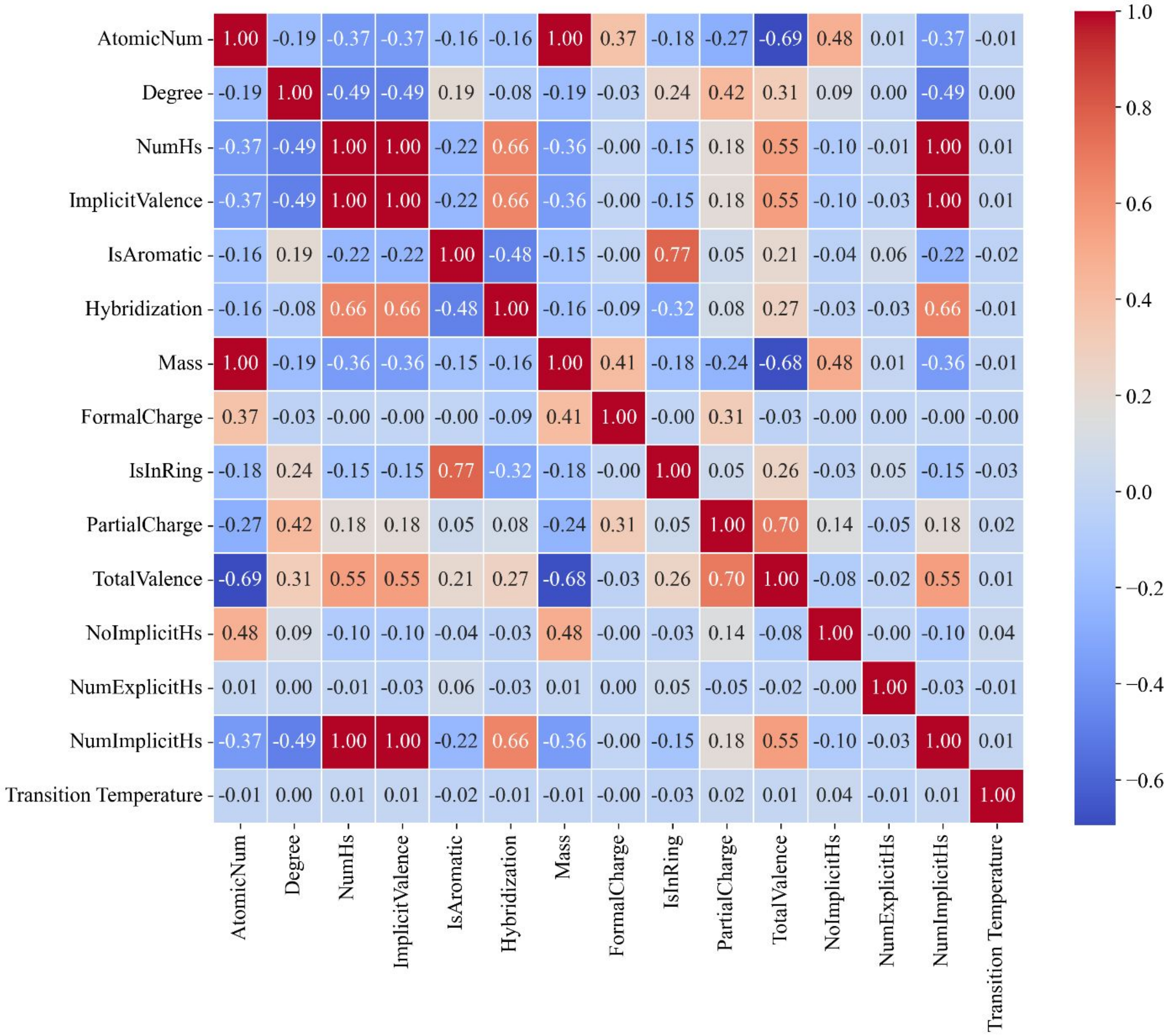

Figure S2. Pearson correlation coefficients among the fourteen atomic features. The values within the matrix represent the correlation strength, where +1 and −1 correspond to perfectly positive and perfectly negative linear relationships, respectively.

Table S3. Molecular descriptors extracted from RDKit and their descriptions.

Feature	Description
**MaxEStateIndex / MinEStateIndex**	Maximum and minimum electrotopological state indices.
**MaxAbsEStateIndex / MinAbsEStateIndex**	Maximum and minimum absolute electrotopological state indices.
**qed**	Quantitative estimate of drug-likeness.
**MolWt / ExactMolWt / HeavyAtomMolWt**	Molecular weights (standard, exact, or heavy atom only).
**NumValenceElectrons**	Total number of valence electrons in the molecule.
**NumRadicalElectrons**	Total number of radical electrons in the molecule.
**MaxPartialCharge / MinPartialCharge**	Maximum and minimum atomic partial charges.
**FpDensityMorgan1/2/3**	Fingerprint density per atom for Morgan fingerprints of radius 1–3.
**BalabanJ**	Balaban's J index (a topological descriptor of molecular connectivity).
**BertzCT**	Bertz complexity index, reflecting molecular structural complexity.
**Chi0–Chi4 (n/v)**	Connectivity indices of order 0–4, with/without valence correction.
**HallKierAlpha**	Hall–Kier alpha value, related to molecular flexibility.
**Ipc**	Information content index.
**Kappa1–Kappa3**	Kier's shape indices describing molecular shape.
**LabuteASA**	Approximate surface area using Labute's method.
**PEOE_VSA1–14**	Van der Waals surface area contributions from PEOE charges (binned).
**SMR_VSA1–10**	Van der Waals surface area contributions from molar refractivity (binned).
**SlogP_VSA1–12**	Van der Waals surface area contributions from logP values (binned).
**TPSA**	Topological polar surface area.
**EState_VSA1–11**	Van der Waals surface area contributions from electrotopological states (binned).
**VSA_EState1–10**	Electrotopological state contributions mapped to surface area (binned).
**FractionCSP3**	Fraction of carbons that are sp3-hybridized.
**HeavyAtomCount**	Number of heavy (non-hydrogen) atoms.
**NHOHCount**	Number of –NH or –OH groups.
**NOCount**	Number of nitrogens and oxygens.
**NumAliphatic/Aromatic/Saturated Rings**	Counts of different ring types in the molecule.

**NumHAcceptors / NumHDonors**	Counts of hydrogen bond acceptors and donors.
**NumHeteroatoms**	Number of heteroatoms in the molecule.
**NumRotatableBonds**	Number of rotatable bonds.
**RingCount**	Total number of rings in the molecule.
**MolLogP**	Octanol–water partition coefficient (logP).
**MolMR**	Molar refractivity.
**BCUT2D_MWLOW**	A BCUT (Burden eigenvalue) descriptor based on molecular weight, reflecting shape and mass distribution of the molecule.
**NumSaturatedCarbocycles**	Number of fully saturated (non-aromatic) carbocyclic rings in the molecule.
**Functional group flags (fr_*)**	Binary descriptors indicating the presence of specific functional groups (e.g., aldehyde, amide, benzene, etc.).
**fr_Al_OH**	Fragment count for aliphatic alcohol groups (–OH attached to non-aromatic carbon).
**fr_Al_OH_noTert**	Similar to above but excludes tertiary alcohol groups.
**fr_bicyclic**	Fragment count for bicyclic systems (two fused or bridged rings).

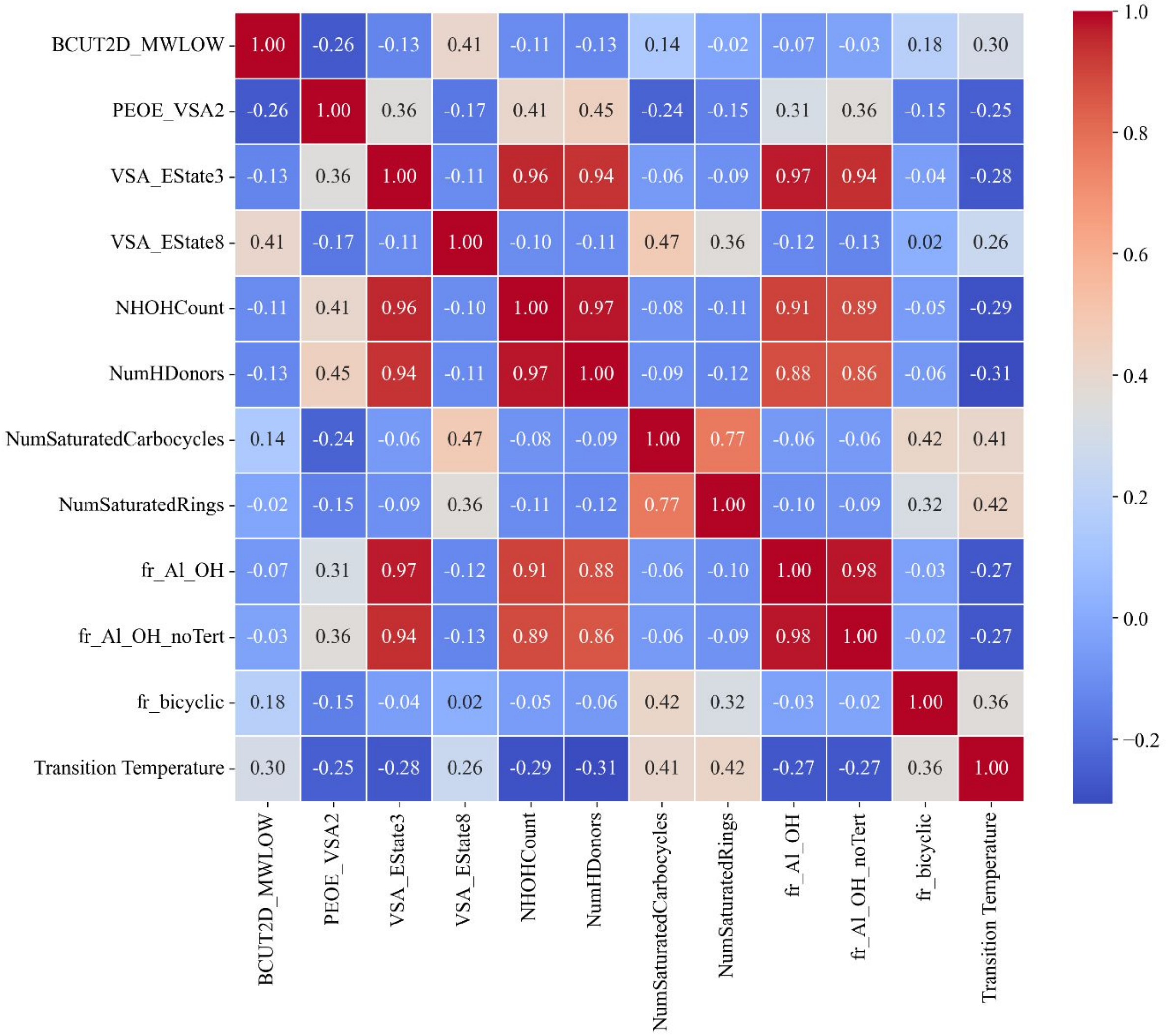

Figure S3. Correlation matrix of the selected molecular descriptors with the transition temperature of the polymers (All molecular-level features and their descriptions are summarized in Table S3).

S1.1. Electronegativity Difference Calculation

To quantify bond polarity, we calculated the absolute difference in Pauling electronegativity ($|\Delta EN|$) between the two bonded atoms. A lookup table of Pauling electronegativities was compiled for the elements most frequently encountered in the dataset (e.g., H, C, N, O, F, Si, P, S, Cl, Br, I, Na, Mg, Al, K, Ca). For each bond, the atomic numbers of the two bonded atoms were retrieved, and their corresponding Pauling electronegativities were obtained from **Table S4**. The bond-level descriptor was then defined as the absolute difference $|EN1 - EN2|$. This feature captures the polarity contribution of bonds, complementing structural descriptors such as bond order, aromaticity, and conjugation.

Table S4. Pauling electronegativity values used for calculating bond polarity features.

Element	Pauling Electronegativity (-)
**H (1)**	2.20
**B (5)**	2.04
**C (6)**	2.55
**N (7)**	3.04
**O (8)**	3.44
**F (9)**	3.98
**Si (14)**	1.90
**P (15)**	2.19
**S (16)**	2.58
**Cl (17)**	3.16
**Br (35)**	2.96
**I (53)**	2.66
**Na (11)**	0.93
**Mg (12)**	1.31
**Al (13)**	1.61
**K (19)**	0.82
**Ca (20)**	1.00

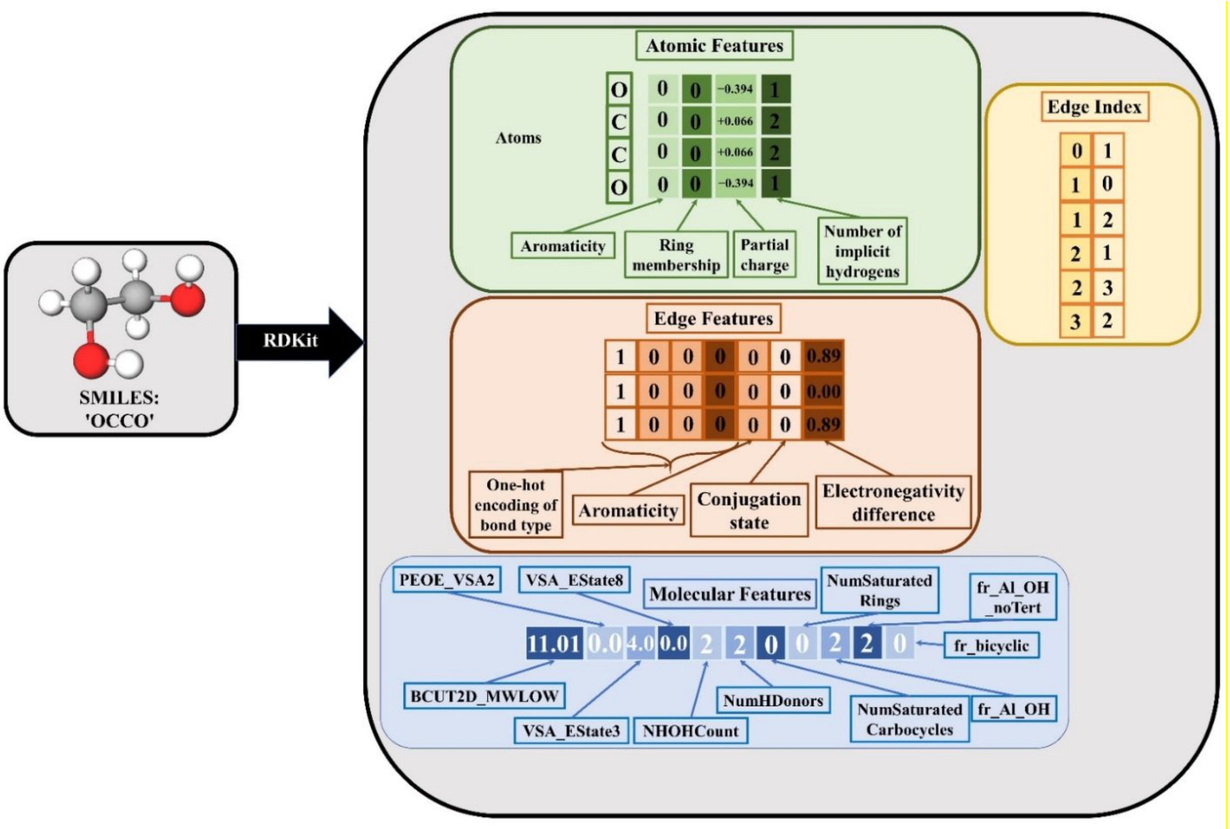

Figure S4. Feature construction for a representative monomer (SMILES: "OCCO") using RDKit. Atomic features (green) encode local properties such as aromaticity, ring membership, partial charge, and number of implicit hydrogens. Bond-level edge features (orange) capture bond type, aromaticity, conjugation state, and electronegativity difference, with connectivity represented by the edge index (yellow). Global molecular descriptors (blue), including PEOE_VSA2, VSA_EState8, BCUT2D_MWLOW, and functional group counts, provide mixture-level information. This hierarchical representation integrates atom-, bond-, and molecule-level attributes as input to the graph neural network.

S2. Hyperparameter Tuning

We performed a latent-dimension sweep $L \in \{16,32,64,128,256,512,1024,2048\}$ using the selected hyperparameters. Validation performance peaked at L=64 with $R^2 \approx 0.8293$; larger dimensions produced no gain and mildly degraded accuracy while increasing computation. Accordingly, we fixed the encoder width to 64 for final training and all downstream analyses (summary in **Table S5**).

Table S5 Performance of the SVR model using latent vectors of different dimensions as input features.

Input Features	Train			Test		
	R^2	MAE	MSE	R^2	MAE	MSE
**16**	0.9490 ± 0.0081	0.0327 ± 0.0013	0.0020 ± 0.0003	0.7431 ± 0.1173	0.0641 ± 0.0183	0.0108 ± 0.0083
**32**	0.9577 ± 0.0031	0.0314 ± 0.0015	0.0016 ± 0.0001	0.8041 ± 0.0772	0.0588 ± 0.0183	0.0083 ± 0.0062
**64**	0.9607 ± 0.0028	0.0305 ± 0.0012	0.0015 ± 0.0001	**0.8293 ± 0.0447**	**0.0546 ± 0.0118**	**0.0069 ± 0.0040**
**128**	0.9589 ± 0.0044	0.0312 ± 0.0017	0.0016 ± 0.0002	0.8242 ± 0.0619	0.0547 ± 0.0142	0.0075 ± 0.0054
**256**	0.9606 ± 0.0031	0.0302 ± 0.0008	0.0015 ± 0.0001	0.8094 ± 0.0862	0.0545 ± 0.0067	0.0070 ± 0.0028
**512**	0.9665 ± 0.0019	0.0288 ± 0.0010	0.0013 ± 0.0001	0.8159 ± 0.0579	0.0560 ± 0.0160	0.0075 ± 0.0046
**1024**	**0.9952 ± 0.0014**	**0.0101 ± 0.0005**	**0.0002 ± 0.0000**	0.7672 ± 0.0852	0.0570 ± 0.0145	0.0098 ± 0.0070
**2048**	0.9569 ± 0.0042	0.0314 ± 0.0016	0.0017 ± 0.0002	0.8050 ± 0.0570	0.0579 ± 0.0136	0.0080 ± 0.0048

Table S6. Effect of virtual-node embeddings on SVR prediction accuracy for polymer transition temperatures.

Models	Train			Test		
	R^2	MAE	MSE	R^2	MAE	MSE
SVR model (With virtual nodes)	0.9607 ± 0.0028	0.0305 ± 0.0012	0.0015 ± 0.0001	**0.8293 ± 0.0447**	**0.0546 ± 0.0118**	**0.0069 ± 0.0040**
SVR model (Without virtual nodes)	0.9632 ± 0.0042	0.0301 ± 0.0012	0.0014 ± 0.0002	0.7662 ± 0.0920	0.0613 ± 0.0176	0.0098 ± 0.0074

Model parameters were learned by minimizing the mean-squared error (MSE) between predictions and experimental transition temperatures. To systematically identify the best-performing model configuration, we employed Bayesian hyperparameter optimization with Optuna. Each trial instantiated a fresh PolyT-GNN model, optimizer, and scheduler, and explored key architectural and training hyperparameters, including number of GNN layers $\{2, 3, 4, 5\}$, dropout rate $(0.3 - 0.5)$, batch size $\{32, 64, 125, 256\}$, and weight decay $(10^{-4} - 10^{-3})$. All models were trained with AdamW at a fixed initial learning rate of $2{\times}10^{-3}$, while a ReduceLROnPlateau scheduler adapted the learning rate based on validation loss. Each trial ran up to 600 epochs with early stopping (patience = 120) and gradient clipping ($\|\nabla\| \leq 5$) to ensure stable convergence. The validation R^2 was reported to Optuna at each epoch, enabling dynamic pruning of underperforming trials. This search strategy allowed efficient exploration of the parameter space, balancing architectural depth and regularization strength. The optimal

configuration selected by Optuna consisted of Five GINEConv layers, dropout 0.30, batch size 256, and weight decay 1×10^{-4}, which was subsequently adopted for the final training and evaluation.

Table S7. Hyperparameter search space and selected configuration.

Hyperparameter	Search Space	Optimal Value (Selected)
**Hidden dimension**	{16, 32, 64, 128, 256, 512, 1024, 2048}	64
**GNN layers**	{2, 3, 4, 5}	5
**Dropout rate**	0.30 – 0.50	0.40
**Batch size**	$\{16, 32, 64, 128, 256\}$	16
**Weight decay**	Log-uniform ($1e^{-4}$ $-$ $1e^{-3}$)	1×10^{-4}
**Start Learning rate**	2×10^{-3}	2×10^{-3}
**Optimizer**	AdamW	AdamW
**Scheduler**	ReduceLROnPlateau (factor=0.5, patience=100)	ReduceLROnPlateau
**Gradient clipping**	Max norm = 5.0	5.0
**Max epochs**	600 (per trial), 2000 (final model)	2000 (final training)
**Early stopping**	Patience = 120 epochs	120

Table S8. Summary of the hyperparameter settings used for the baseline machine learning models in predicting the transition temperatures of 2W-SMPs.

Model	Hyperparameter	Value/Setting
Gaussian Process Regression (GPR)	Constant kernel amplitude	1
	RBF length scale	1
	White noise level	0.05
Support Vector Regression (SVR)	Kernel function	Radial Basis Function (RBF)
	Regularization parameter	1.1721
	Epsilon	0.03
	RBF kernel width	0.0386
Kernel Ridge Regression (KRR)	Regularization coefficient	0.00187
	Kernel function	Radial Basis Function (RBF)
	RBF kernel width	0.01918

S3. Silhouette coefficient

For each datapoint (monomer) i, the Silhouette value $s(i)$ is defined as:

$$s(i) = \frac{b(i) - a(i)}{\max\{a(i), b(i)\}} \quad \text{(S1)}$$

where, $a(i)$ is the average distance between point i and all other points in the same clusters:

$$a(i) = \frac{1}{|C_i|} \sum_{j \epsilon C_i, j \neq i} d(i,j) \quad \text{(S2)}$$

here, C_i is the cluster containing i, and $d(i,j)$ is typically the Euclidean distance between latent embeddings. Moreover, $b(i)$ is the minimum average distance between point i and all points in the nearest neighboring cluster (inter-cluster dissimilarity).

$$b(i) = \underbrace{min}_{k \neq C_i} \frac{1}{|C_i|} \sum_{j \epsilon C_k} d(i,j) \quad \text{(S3)}$$

The overall silhouette score for a dataset of N points is the mean of all individual values:

$$S = \frac{1}{N} \sum_{i=1}^{N} s(i) \quad \text{(S4)}$$

To compute the Silhouette coefficient, latent representations of all monomers obtained from the encoder were grouped using K-means clustering with the number of clusters set to three. Each monomer was thus assigned to one of three unsupervised clusters in the latent space, from which the intra- and inter-cluster distances $a(i)$ and $b(i)$ were calculated. The choice of three clusters is physically motivated by the three primary switching-domain types governing reversible actuation in 2W-SMPs: (i) semicrystalline systems dominated by melting/crystallization transitions (T_{m}), (ii) amorphous systems actuated by glass transition (T_{g}), and (iii) liquid-crystalline systems controlled by the nematic–isotropic transition (T_{ni}). This selection provides a

chemically interpretable structure to the latent manifold and enables quantitative evaluation of how well the fine-tuned latent space differentiates among these actuation mechanisms.

S4. Maximum Mean Discrepancy (MMD)

The Maximum Mean Discrepancy (MMD) quantifies the distance between two probability distributions (the latent distributions of general polymers and 2W-SMPs). Given two sets of samples:

$$X = \{x_1, x_2, \ldots, x_m\} \sim P, \qquad Y = \{y_1, y_2, \ldots, y_n\} \sim Q \tag{S5}$$

MMD is defined as:

$$MMD(X,Y) = \sqrt{\frac{1}{m^2}\sum_{i=1}^{m}\sum_{j=1}^{m} k(x_i,x_j) + \frac{1}{n^2}\sum_{i=1}^{n}\sum_{j=1}^{n} k(y_i,y_j) - \frac{2}{mn}\sum_{i=1}^{m}\sum_{j=1}^{n} k(x_i,y_j)} \tag{S6}$$

where, $k(\cdot,\cdot)$ is a positive-definite kernel function:

$$k(x,y) = \exp\left(-\frac{||x-y||^2}{2\sigma^2}\right) \tag{S7}$$

In this work, a Gaussian kernel with a fixed bandwidth of $\sigma = 1.0$ was used, corresponding to the latent-space scale employed in the similarity computations.

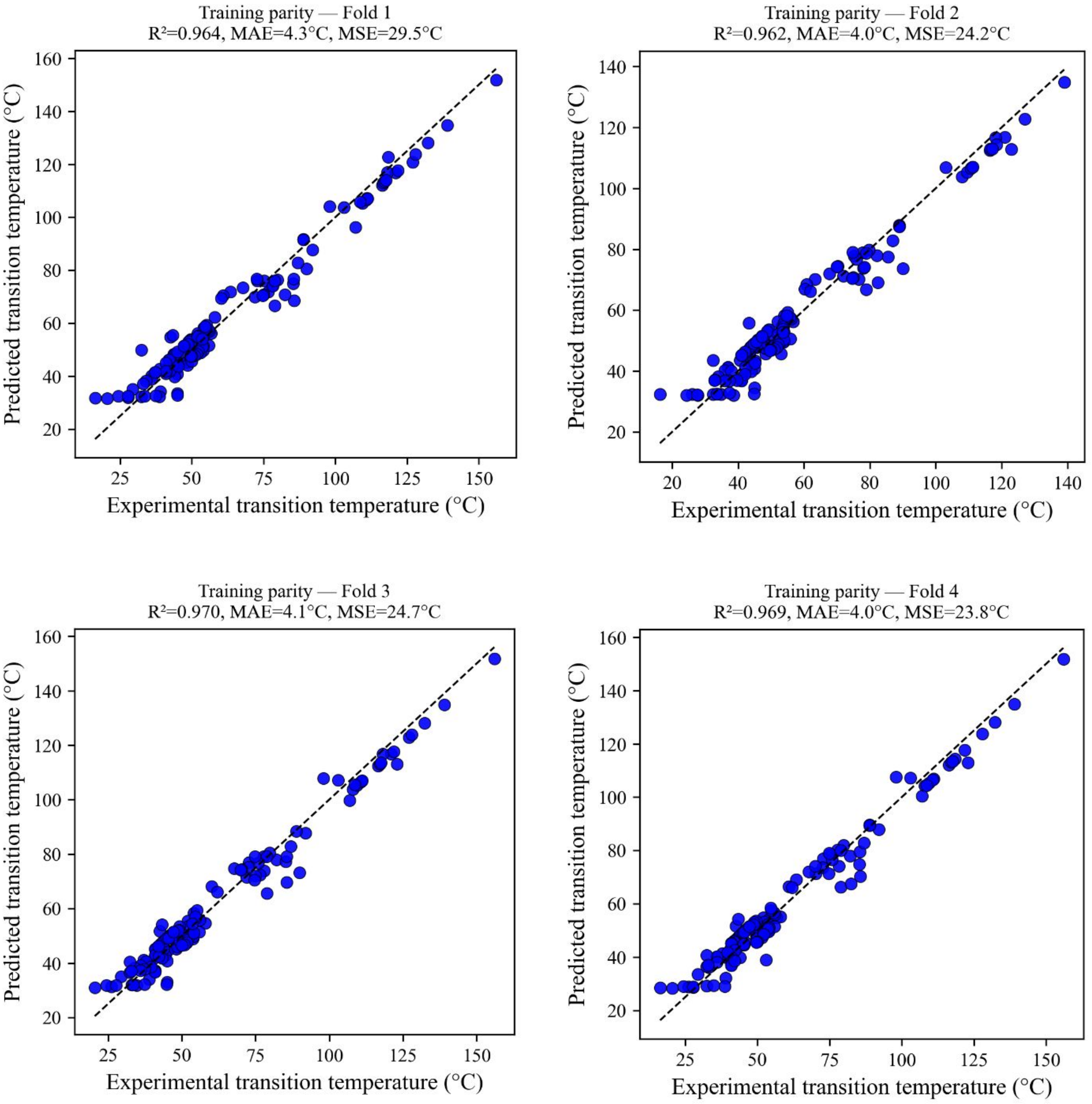
Training parity — Fold 1
R²=0.964, MAE=4.3°C, MSE=29.5°C
Predicted transition temperature (°C)
Experimental transition temperature (°C)
Training parity — Fold 2
R²=0.962, MAE=4.0°C, MSE=24.2°C
Predicted transition temperature (°C)
Experimental transition temperature (°C)
Training parity — Fold 3
R²=0.970, MAE=4.1°C, MSE=24.7°C
Predicted transition temperature (°C)
Experimental transition temperature (°C)
Training parity — Fold 4
R²=0.969, MAE=4.0°C, MSE=23.8°C
Predicted transition temperature (°C)
Experimental transition temperature (°C)

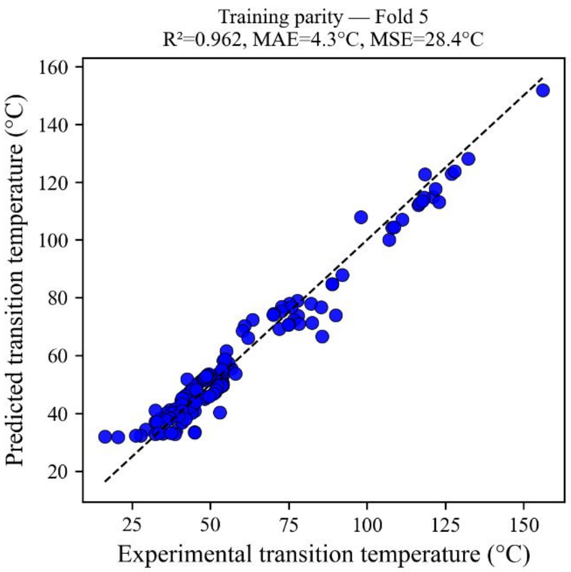

Figure S5. Parity plots of predicted versus experimental transition temperatures for the training data across five-fold cross-validation. Each panel corresponds to one training fold, demonstrating the strong agreement between predicted and experimental values, with all folds exhibiting high R^2 and low MAE and MSE.

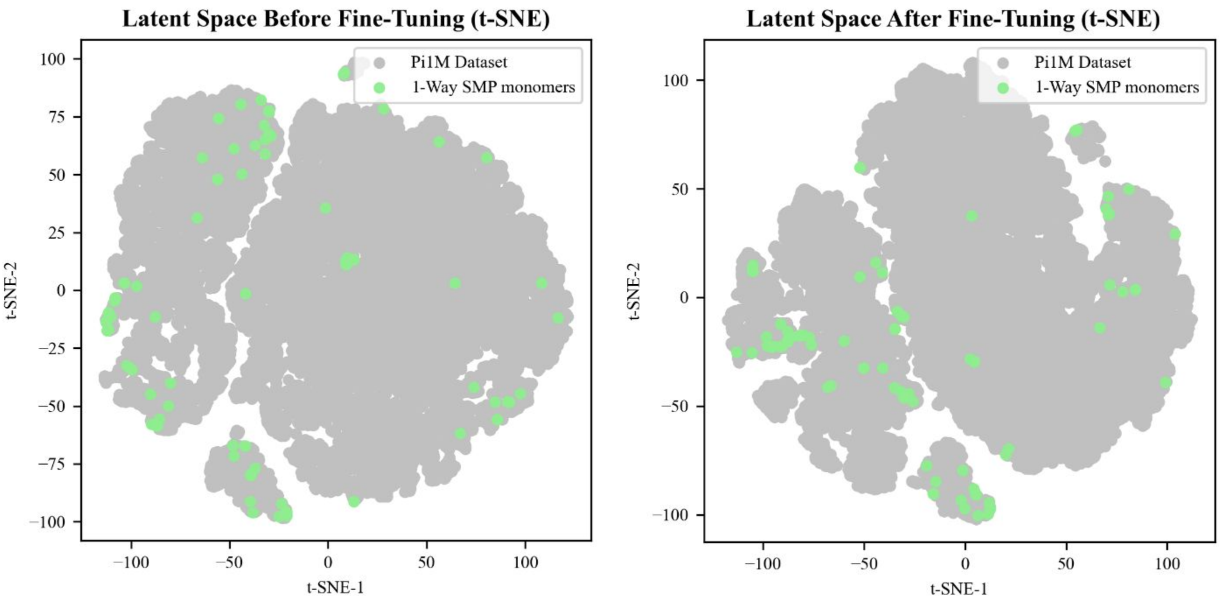

(a) (b)

Figure S6. t-SNE visualization of the latent space before (a) and after (b) fine-tuning on the one-way SMP dataset. Gray points represent polymers from the Pi1M dataset, and green points denote 1W-SMP monomers.

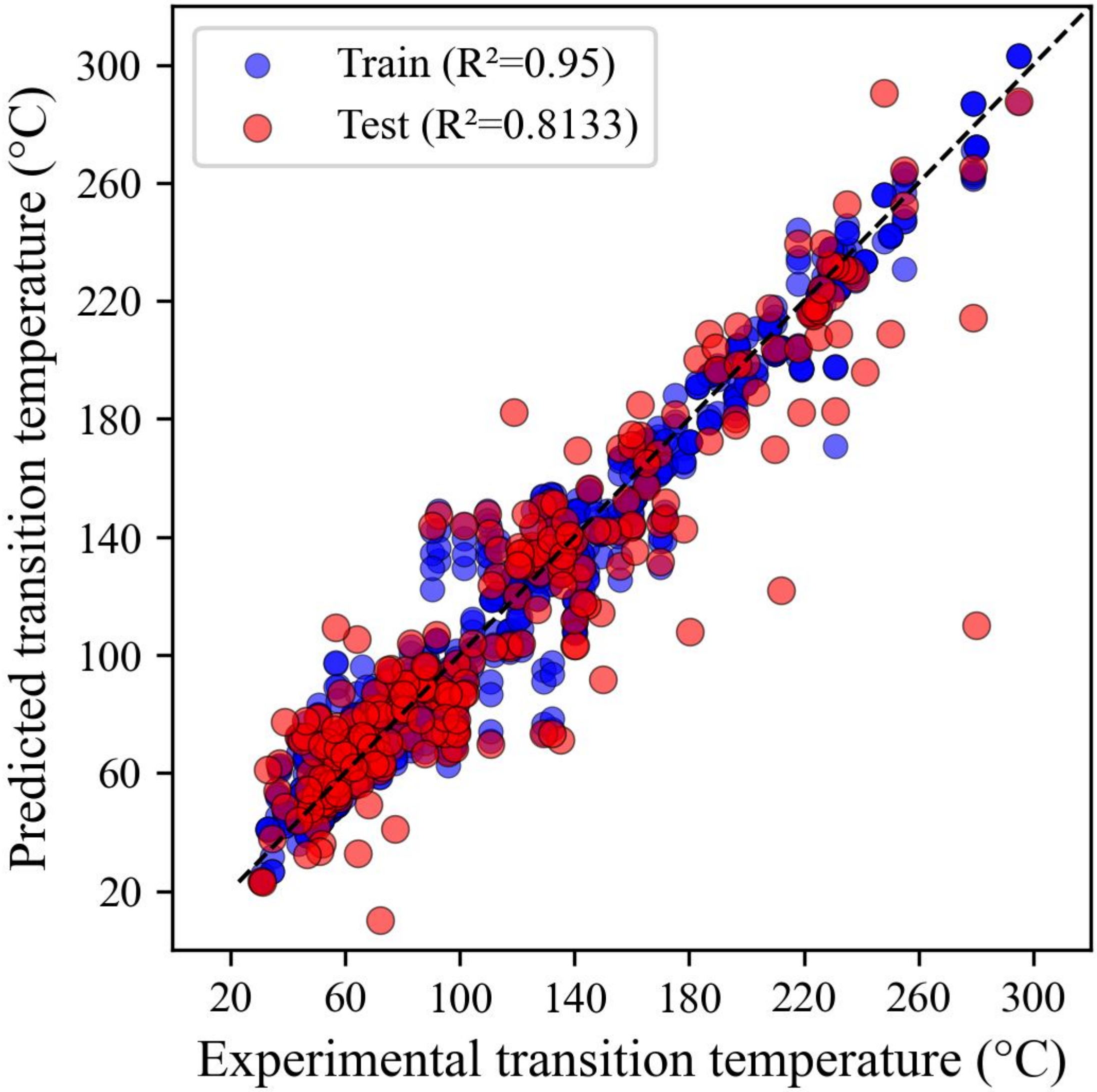

Figure S7. Parity plot of predicted versus experimental transition temperatures for 1W-SMPs, including both training (blue) and test (red) datasets. Training points are obtained from in-sample predictions across five-fold cross-validation, while test points correspond to an independent hold-out set. The close alignment of both datasets with the ideal diagonal line indicates strong predictive performance and good generalization of the model.

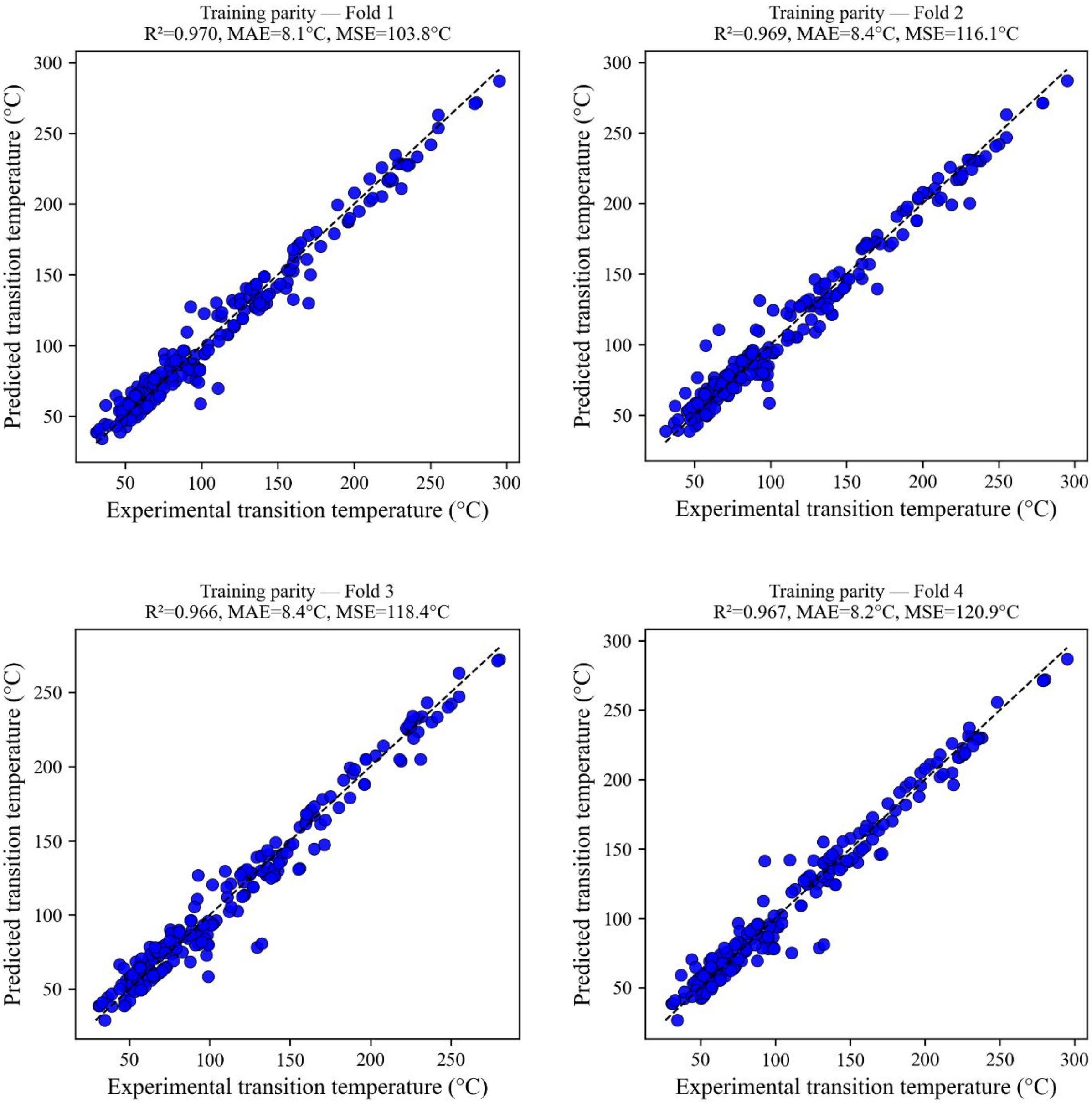
Training parity — Fold 1
R²=0.970, MAE=8.1°C, MSE=103.8°C
Training parity — Fold 2
R²=0.969, MAE=8.4°C, MSE=116.1°C
Training parity — Fold 3
R²=0.966, MAE=8.4°C, MSE=118.4°C
Training parity — Fold 4
R²=0.967, MAE=8.2°C, MSE=120.9°C
Predicted transition temperature (°C)
Experimental transition temperature (°C)
50
100
150
200
250
300

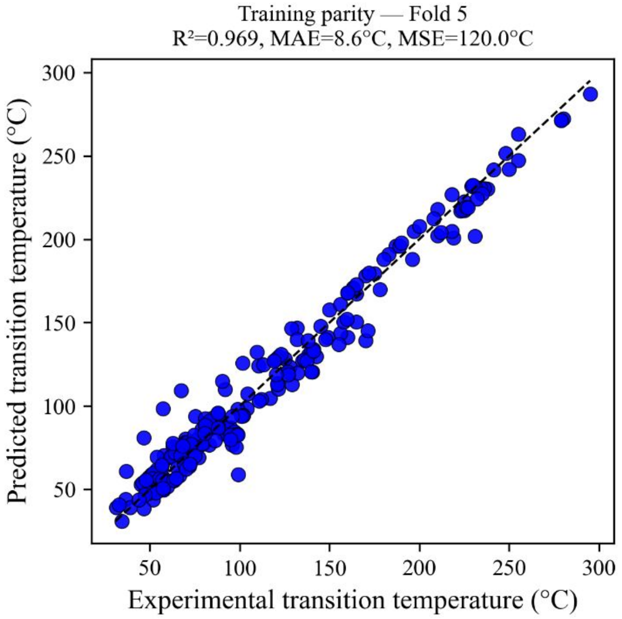

Figure S8. Parity plots of predicted versus experimental transition temperatures for the one-way SMP dataset across five-fold cross-validation. Each panel corresponds to one training fold, demonstrating the strong agreement between predicted and experimental values, with all folds exhibiting high coefficients of determination (R^2) and low prediction errors (MAE and MSE).

To quantitatively evaluate the effect of fine-tuning on the latent representations of one-way SMPs, the Silhouette coefficient and MMD were calculated (**Table S9**). The Silhouette coefficient increased from 0.379 to 0.418 ($\Delta = +0.039$), indicating that the embeddings of one-way SMPs became more compact and better separated from each other after fine-tuning. Meanwhile, the MMD decreased from 0.1533 to 0.1292 ($\Delta = -0.0241$), reflecting a closer alignment between the one-way SMP latent distribution and the broader Pi1M polymer manifold. Together, these metrics confirm that fine-tuning improves both the internal organization of the one-way SMP latent space and its integration with the general polymer representation learned during pretraining.

Table S9. Unsupervised cluster quality and distribution distance of the 1W-SMP latent space before and after fine-tuning.

Metric	Before Fine-Tuning	After Fine-Tuning	Δ (Change)
Silhouette	0.379	0.418	+0.039
MMD	0.1533	0.1292	-0.0241
